# Inhibition of Fried Meat-Induced Colorectal DNA Damage and Altered Systemic Genotoxicity in Humans by Crucifera, Chlorophyllin, and Yogurt

**DOI:** 10.1371/journal.pone.0018707

**Published:** 2011-04-25

**Authors:** Daniel T. Shaughnessy, Lisa M. Gangarosa, Barbara Schliebe, David M. Umbach, Zongli Xu, Beth MacIntosh, Mark G. Knize, Peggy P. Matthews, Adam E. Swank, Robert S. Sandler, David M. DeMarini, Jack A. Taylor

**Affiliations:** 1 Laboratory of Molecular Carcinogenesis, National Institute of Environmental Health Sciences, National Institutes of Health (NIH), Department of Health and Human Services (DHHS), Research Triangle Park, North Carolina, United States of America; 2 Department of Medicine, School of Medicine, University of North Carolina, Chapel Hill, North Carolina, United States of America; 3 Biostatistics Branch, National Institute of Environmental Health Sciences, National Institutes of Health (NIH), Department of Health and Human Services (DHHS), Research Triangle Park, North Carolina, United States of America; 4 Epidemiology Branch, National Institute of Environmental Health Sciences, National Institutes of Health (NIH), Department of Health and Human Services (DHHS), Research Triangle Park, North Carolina, United States of America; 5 Clinical and Translational Research Center, University of North Carolina, Chapel Hill, North Carolina, United States of America; 6 Chemistry, Materials, and Life Sciences Division, Lawrence Livermore National Laboratory, Livermore, California, United States of America; 7 National Health and Environmental Effects Research Laboratory, U.S. Environmental Protection Agency, Research Triangle Park, North Carolina, United States of America; University of California, Los Angeles, and Cedars-Sinai Medical Center, United States of America

## Abstract

Dietary exposures implicated as reducing or causing risk for colorectal cancer may reduce or cause DNA damage in colon tissue; however, no one has assessed this hypothesis directly in humans. Thus, we enrolled 16 healthy volunteers in a 4-week controlled feeding study where 8 subjects were randomly assigned to dietary regimens containing meat cooked at either low (100°C) or high temperature (250°C), each for 2 weeks in a crossover design. The other 8 subjects were randomly assigned to dietary regimens containing the high-temperature meat diet alone or in combination with 3 putative mutagen inhibitors: cruciferous vegetables, yogurt, and chlorophyllin tablets, also in a crossover design. Subjects were nonsmokers, at least 18 years old, and not currently taking prescription drugs or antibiotics. We used the *Salmonella* assay to analyze the meat, urine, and feces for mutagenicity, and the comet assay to analyze rectal biopsies and peripheral blood lymphocytes for DNA damage. Low-temperature meat had undetectable levels of heterocyclic amines (HCAs) and was not mutagenic, whereas high-temperature meat had high HCA levels and was highly mutagenic. The high-temperature meat diet increased the mutagenicity of hydrolyzed urine and feces compared to the low-temperature meat diet. The mutagenicity of hydrolyzed urine was increased nearly twofold by the inhibitor diet, indicating that the inhibitors enhanced conjugation. Inhibitors decreased significantly the mutagenicity of un-hydrolyzed and hydrolyzed feces. The diets did not alter the levels of DNA damage in non-target white blood cells, but the inhibitor diet decreased nearly twofold the DNA damage in target colorectal cells. To our knowledge, this is the first demonstration that dietary factors can reduce DNA damage in the target tissue of fried-meat associated carcinogenesis.

**Trial Registration:**

ClinicalTrials.gov NCT00340743.

## Introduction

Colorectal cancer is the fourth most common cancer worldwide [1], and consumption of red and processed meat has been associated with increased risk of and mortality from this cancer [2], [3]. In particular, consumption of red meat and meat cooked at high temperature containing elevated levels of heterocyclic amines (HCAs) is associated with increased risk of colorectal adenoma [4]. HCAs are mutagenic and carcinogenic compounds formed through pyrolysis of aromatic amino acids and creatinine in meats cooked at high temperature, particularly by pan-frying [5].

Numerous studies *in vitro*, in animals, and in humans indicate that various dietary components, such as cruciferous vegetables, chlorophyllin (CHL), and fermented milk products, may modulate cancer risks associated with meat consumption in general and the mutagenic and carcinogenic effects of HCAs specifically [6]–[11]. Although several small observational studies in humans [6]–[12] reported protective effects of cruciferous vegetables against fried-meat-induced genotoxicity, epidemiologic studies [13] did not. Glucosinolates and isothiocyanates in cruciferous vegetables inhibit HCA-induced genotoxicity by several mechanisms, including inhibition of phase I metabolizing enzymes, induction of phase II detoxification enzymes, and apoptosis [6].

CHL, a copper salt derivative of chlorophyll, reduces the mutagenicity and carcinogenicity of HCAs *in vitro* and in experimental animals; it also reduces the genotoxic effects of aflatoxin exposure in humans [9]. CHL forms a molecular complex with planar carcinogens, thus inhibiting uptake in the intestine [9]; it also exhibits antioxidant activity [14] and induces apoptosis [15].

Animal studies and small controlled feeding studies in humans [8], [16] reported that lactobacilli in fermented milk and yogurt protect against HCA-induced genotoxicity and carcinogenicity. Lactobacilli from dietary sources may inhibit HCA-induced genotoxicity by binding mutagens to the bacterial cell wall or by altered metabolism of HCAs through changes in intestinal microflora [8].

Previous controlled feeding studies in humans focused on changes in urinary mutagenicity after consumption of fried meat or inhibitors of fried meat-induced mutagenesis [17]. Although urine mutagenicity can reflect systemic exposure to dietary mutagens and antimutagens, it does not measure the ability of fried meat to induce DNA damage in relevant cancer-target tissues, such as in colon epithelial cells, or the ability of putative dietary antimutagens and anticarcinogens to reduce such damage.

**Figure 1:**
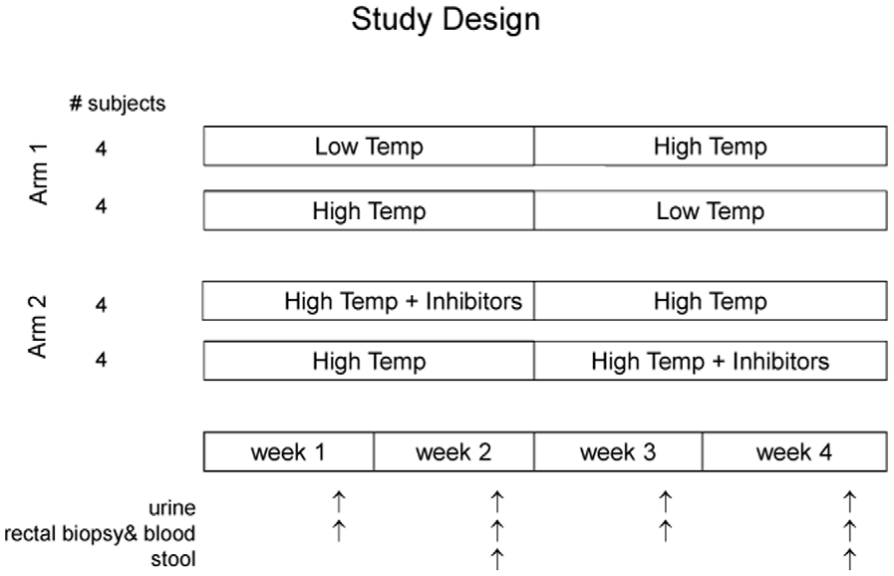
Study Design. Subjects were assigned randomly to arms and to treatment sequences with the restriction that each sequence included 2 men and 2 women. Diets in Arm 1 included meat cooked at low (100°C) and high temperature (250°C); diets in Arm 2 included meat cooked at high temperature either with or without three inhibitors: cruciferous vegetables, yogurt, and chlorophyllin tablets. Subjects consumed each diet in their assigned sequence for two-week periods. Arrows indicate sampling times for urine, stool, rectal biopsies, and leukocytes.

To explore these issues, we used a crossover design and fed subjects diets containing meat fried at low or high temperature (Fig. 1) cooked as described by Sinha et al. [18]. Subjects were also fed diets prepared with meat fried at high temperature alone or in combination with three putative inhibitors of HCA-induced damage (cruciferous vegetables, chlorophyllin tablets, and yogurt), again in a crossover design. Based on the protocol of Peters et al. [17], we evaluated the effects of the cooking methods and diets on meat and urinary mutagenicity using the *Salmonella* (Ames) mutagenicity assay, and we also extended this to fecal mutagenicity. To assess the effects of the diets on target and non-target tissue, we used the single cell gel electrophoresis (comet) assay to measure DNA damage in epithelial cells isolated from rectal biopsies and from lymphocytes isolated from peripheral blood. To our knowledge this is the first study in humans to combine measurements of fecal and urinary mutagenicity with assessment of DNA damage in the target tissue, colon epithelium, to evaluate the genotoxic effects of HCAs and inhibition of that genotoxicity by dietary factors, *i.e.*, cruciferous vegetables, CHL, and yogurt.

## Results

### Subject recruitment

Of the 70 individuals assessed for eligibility, 24 did not meet the inclusion criteria and 30 declined to participate. Individuals were excluded for weight (BMI>31), diet, age, underlying medical conditions, prescription medication use, or NSAID use. Subjects were recruited between April and August, 2004 and participated in the study in 4 groups of 4 between June and November, 2004. All 16 subjects enrolled in the study completed the study protocol and results were collected and analyzed for all subjects.

### Mutagenicity of meat extracts

Before the feeding study, we evaluated mutagenicity in a variety of meats cooked at high and low temperature; results of this pilot study (Table 1) were consistent with previous reports [17], [18]. The extract from beef cooked at low temperature was not mutagenic and had non-detectable levels of HCAs. In contrast, the extract from beef cooked at high temperature had high levels of HCAs, with PhIP (2-amino-1-methyl-6-phenylimidazo[4,5-*b*]pyridine) being predominant, and the extract was mutagenic, exhibiting an ∼10-fold higher mutagenic potency in strain YG1024 compared to strain TA98. The results with sausage were similar to those with high-temperature beef, and extracts from both meats were less mutagenic in YG1041 compared to YG1024.

**Table 1:** Estimated mutagenicity of fried meats from pilot study.

		Mutagenicity (rev/g-eq)^a^		
Meat	Temp.	TA98	YG1024	YG1041
Beef	Low	8.5±9.5	−1.0±11.9	8.3±14.8
	High	1057±307^b^	10095±916^b^	8600±169
Sausage	Low	Not tested	Not tested	Not tested
	High	904±81	6910±194	2782±61
Bacon	Low	Not tested	Not tested	Not tested
	High	3759±253	26649±631	16877±526

a Unless otherwise noted, each estimate is based on one preparation (meat sample) assayed once; standard error reflects only variation within the plate-incorporation assay.

b Estimate is based on three independent preparations (meat samples) each assayed once; standard error reflects variation among samples, among assays, and within assays.

The extract from high-temperature bacon had extremely high levels of HCAs and mutagenicity and was 4.5 and 7 times more mutagenic in YG1041 and YG1024, respectively, than in TA98. Although the extract of beef was similarly mutagenic in YG1024 and YG1041, the presence of nitroreductase in YG1041 clearly reduced the mutagenicity of extracts from sausage and bacon, which likely contained nitrites that helped to form nitroarenes that were converted to aromatic amines by nitroreductase in YG1041. The potentially high nitrite levels in bacon may account for the extremely high levels of mutagenicity of bacon extracts in all three strains. Because of the potential contribution of nitrites along with HCAs with the bacon, we decided not to include bacon in the feeding study so that we were examining the effect of primarily HCAs.

Mean levels of HCAs and mutagenicity of extracts from 4 batches of high-temperature beef and sausage prepared throughout the feeding study (Table 2) were consistent with those from the pilot, with PhIP being the predominant HCA and both extracts exhibiting similar levels of mutagenic activity, ∼10,000 rev/g in YG1024.

**Table 2:** Mean HCA levels and mutagenicity of low- and high-temperature-fried meats.

		HCAs (ng/g)				YG1024
Meat	Temperature	MeIQx	PhIP	DiMeIQx	IFP	(rev/g-eq)
Beef^a^	Low	0	0.105±0.005	0	0	−1.0±11.9
			(0.041, 0.169)			(−24.4, 22.4)
Sausage^a^	Low	0	0	0	0	Not tested
Beef^b^	High	10.6±2.6	67.3±24.6	4.1±0.9	11.6±3.0	11569±3625
		(2.5, 18.8)	(−11.0, 145.7)	(1.1, 7.0)	(2.1, 21.1)	(1503, 21635)
Sausage^b^	High	13.4±2.0	33.3±11.6	4.0±0.7	10.5±2.2	10151±3624
		(7.1, 19.6)	(−3.7, 70.3)	(1.8, 6.2)	(3.3, 17.7)	(90, 20212)

a Estimate ± S.E. (95% confidence interval) based on 1 meat sample from the pilot study; standard errors reflect variation among duplicate assays for HCAs and variation within a single plate-incorporation assay for mutagenicity.

b Estimate ± S.E. (95% confidence interval) based on 4 meat samples, each assayed once from the feeding study; standard error reflects variation among samples, among assays, and within assays.

### Urinary mutagenicity

**Figure 2:**
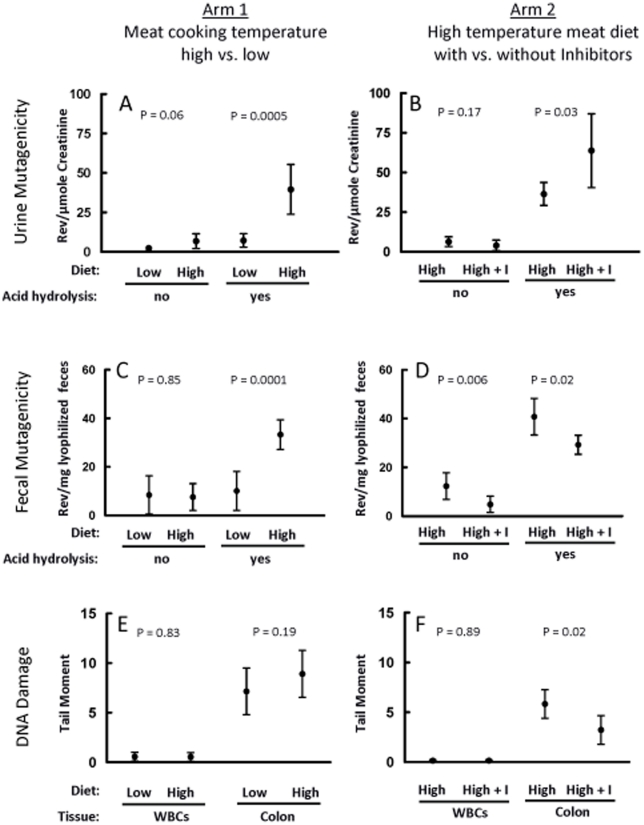
Dietary effects on urine and fecal mutagenicity and on DNA damage. Each arm had 8 subjects who consumed both diets in a crossover design (Fig. 1); thus each estimate reflects data from 8 subjects. Error bars represent 95% confidence limits of the estimates calculated through the fitted mixed-model and include subject-to-subject variation whereas *P*-values for diet comparisons utilize smaller within-subject variation. Low = diet with meat fried at low temperature (100°C); High = diet with meat fried at high temperature (250°C); High+I = diet with meat fried at high temperature and inhibitors. Arm 1: Low versus High; Arm 2: High versus High+I. Panels (A) and (B): Estimated mean mutagenicity (rev/µmole creatinine) of hydrolyzed and un-hydrolyzed urine extracts for Arm 1 and Arm 2, respectively. Mutagenicity is adjusted for the creatinine level in each sample. Panels (C) and (D): Estimated mean mutagenicity (rev/mg lyophilized feces) of hydrolyzed and un-hydrolyzed fecal extracts for Arm 1 and Arm 2, respectively. Panels (E) and (F): Estimated mean levels of DNA damage assessed by the comet assay in WBCs and colon epithelial cells for Arm 1 and Arm 2, respectively, based on the average across 4 slides per sample of median Tail Moment on 50 cells per slide.

The estimated mutagenic potencies of extracts from un-hydrolyzed and hydrolyzed urine from 8 subjects depended on whether they consumed meals prepared with meat cooked at low or high temperature (Fig. 2A). Urinary mutagenicity in un-hydrolyzed samples increased nearly 3-fold (*P* = 0.06), from 2.3 to 6.8 rev/µmol creatinine, respectively, from low- to high-temperature meat diets. The mutagenic potency of extracts from hydrolyzed urine increased significantly (*P* = 0.0005) from 7.2 to 39.6 rev/µmol creatinine, respectively, from low- to high-temperature meat diets. These results were consistent with the high levels of HCAs measured in high-temperature meat.

For the 8 subjects consuming diets containing high-temperature meat alone or in combination with the dietary inhibitors, the mutagenic potency of extracts from un-hydrolyzed urine decreased slightly when subjects consumed the inhibitors (Fig. 2B). In contrast, the mutagenic potency of extracts from hydrolyzed urine increased nearly 2-fold, from 36.5 to 63.8 rev/µmol creatinine, respectively, with the addition of inhibitors to the high-temperature meat diet (*P* = 0.03).

### Fecal mutagenicity

The % extractable organic matter (EOM) ± SD for the feces was 8.4±1.7 for un-hydrolyzed, 8.7±1.9 for hydrolyzed, and 8.6±1.8 for the two combined. We saw no difference in fecal mutagenicity in extracts from un-hydrolyzed feces between low- and high-temperature meat diets (Fig. 2C). In contrast, extracts of hydrolyzed feces from subjects eating the high-temperature meat diet were significantly more mutagenic (*P*<0.0001) than those from subjects eating the low-temperature meat diet, being, respectively, 33.3 versus 10.1 rev/mg of lyophilized feces. Adding inhibitors to the high-temperature meat diet decreased the mutagenic potencies of extracts compared to the diet without inhibitors (Fig. 2D) from 12.3 to 4.9 rev/mg of lyophilized feces for un-hydrolyzed feces (*P* = 0.006) and from 40.8 to 29.3 rev/mg of lyophilized feces for hydrolyzed feces (*P* = 0.019).

### DNA damage in colon cells and WBCs

The mean comet-assay Tail Moment (TM) of colon cells was 7.2 with low- and 8.9 with high-temperature meat, but the increase was not statistically significant (*P* = 0.19) (Fig. 2E–F). When subjects consumed dietary inhibitors along with the high-temperature meat, DNA damage in colon cells was reduced significantly by nearly half, from a mean TM of 5.8 without inhibitors to 3.2 with inhibitors (*P* = 0.02). We saw no differences among diets for DNA damage in WBCs (Fig. 2E–F).

## Discussion

### HCAs and mutagenicity of meat extracts

In our study, high-temperature beef and sausage had HCA levels (Table 2) comparable to those of Sinha et al. [18] who reported concentrations of 9, 2.1, and 32.8 ng/g of meat for MeIQ (2-amino-3,8-dimethylimidazo[4,5-*f*]quinoxaline), DiMeIQx (2-amino-3,4,8-trimethylimidazo[4,5-*f*]quinoxaline), and PhIP, respectively. For the predominant HCA, PhIP, our subjects received ∼4 g of meat/kg/day×50.3 ng of PhIP/g of meat = 201.2 ng of PhIP/kg/day, corresponding to 118 ng/kg/day in Sinha et al. [18].

Based on the mutagenicity of high-temperature beef and sausage in strain TA98 from our pilot study (Table 1), our subjects received ∼1000 rev/g of meat×∼4 g/kg/day = 4000 rev/kg/day. Our subjects averaged 86 kg in weight, so a typical subject had an intake of ∼344,000 rev/day from high-temperature meat; Peters et al. [17] reported 253,700 rev/day in a study with a similar protocol. Extracts from fried beef and sausage were 10 times more mutagenic in YG1024 than in TA98 (Table 1), confirming a previous study [19], and was likely due to activation of HCAs by acetyltransferase in YG1024. When based instead on meat-derived mutagenicity measured in strain YG1024, our subjects had an intake of ∼3,440,000 rev/day.

### Effect of fried meat on urinary and fecal mutagenicity

By design this was not a metabolic study; nevertheless, we constructed approximations to a typical subject's output of mutagenicity (Table 3). Because our samples include only a portion of the urine or feces eliminated by a subject on a given day, our approximations likely underestimate output. The increase in HCAs in the meat was associated with a concomitant increase in urinary mutagenicity from low- to high-temperature meat diets, corroborating a previous finding [17] (Table 3). Unlike the previous study [17], we used the same strain of *Salmonella* (YG1024) to measure the input of meat-derived mutagenicity and the output of urinary (and fecal) mutagenicity. Our approximations indicate that subjects on the high-temperature meat diet excreted 3 and 16% of ∼3,440,000 rev/day intake as unhydrolyzed and hydrolyzed urinary mutagenicity, respectively (Table 3).

**Table 3:** Approximate output of urinary (rev/12 h) and fecal (rev/movement) mutagenicity.

Source	Study	Meat temperature	Un-hydrolyzed	Hydrolyzed
Urine	Peters et al. (2004)	Low	4, 537^a^	51,527^b^
		High	101,181^a^	399,582^b^
	This study^c^	Low	32,000	101,000
		High	95,000	553,000
		High−inhibitors	89,000	509,000
		High+inhibitors	57,000	891,000
Feces	This study^d^	Low	163,000	196,000
		High	147,000	643,000
		High−inhibitors	238,000	787,000
		High+inhibitors	94,000	565,000

a Mean for 10 subjects.

b Mean for 60 subjects.

c Values converted from mean mutagenic potencies in rev/µmole creatinine (Fig. 2A and 2B) assuming 13964 µmole creatinine per 12 h urine sample. This conversion factor was a median based on 36 samples (4 samples each for 8 subjects; 1 sample each for 4 subjects) of first morning voids. Although we measured creatinine concentrations for all 64 samples (4 samples each from 16 subjects), we failed to record urine volume for 28 (4 samples each for 4 subjects plus 3 samples each for 4 subjects).

d Values converted from mean mutagenic potencies in rev/mg lyophilized feces (Fig. 2C and 2D) assuming 19.32 g lyophilized feces per movement. This conversion factor was a median based on 32 fecal samples (2 samples each for 16 subjects).

Hydrolyzed urinary mutagenicity reflects both the un-conjugated and formerly conjugated mutagenic activity. One study [20], however, showed an association between risk for colorectal adenomas and unhydrolyzed urinary mutagenicity. Interestingly, the diet with inhibitors reduced the excretion of unhydrolyzed urinary mutagenicity by 36% (Table 3) compared to the same diet without them, suggesting that inhibitors reduced the systemic genotoxic exposure (*i.e.*, unhydrolyzed urinary mutagenicity) associated with risk for colorectal adenoma. The approximations in Table 3 indicate that without inhibitors, ∼38% of the meat-derived mutagenic intake was excreted (hydrolyzed fecal and urinary mutagenicity combined), *i.e.*, 509,000+787,000 = 1,296,000 rev/day excreted versus 3,440,000 rev/day consumed; with inhibitors present, this value was ∼42%. We suspect that additional mutagenic activity would have been revealed by capturing all urine and feces excreted and by fractionating the feces [21]. There are several classes of fecal mutagens [22], and some, such as the fecapentanes, were not necessarily measured in our study because they are primarily direct-acting, base-substitution mutagens, and we used a frameshift strain with S9 mix.

Consumption of meat fried at high temperature increased levels of hydrolyzed fecal mutagenicity, consistent with a previous study [21] showing that consumption of fried meat increased fecal mutagenicity in humans. Also, fried-meat mutagens in feces were in primarily a conjugated (or bound) form. HCAs bind to intestinal bacteria *in vitro* [23], and dietary fiber (non-starch polysaccharides) reduces HCA bioavailability *in vivo* [24]. Thus, meat-derived HCAs may have been bound to dietary fiber and normal gut flora, and their mutagenicity was revealed only when they were released by acid hydrolysis. Because the total dietary fiber content was approximately 38% greater with inhibitors than without them, we cannot rule out a protective role for fiber in the inhibitor diet.

### Effect of inhibitors on urinary and fecal mutagenicity

Although the diet containing inhibitors produced a non-significant decrease in un-hydrolyzed urinary mutagenicity, it increased hydrolyzed urinary mutagenicity nearly 2-fold (Fig. 2B). This increase was likely due to enhanced conjugation, possibly by components of the crucifera [6]. Cruciferous vegetables induced the phase II enzyme NAD(P)H quinone reductase *in vitro* [25], induced UDP-glucuronosyl transferase in rats [26], and increased glutathione-*S*-transferase-α levels in human plasma and colon epithelium [27]–[29].

The decrease in both hydrolyzed and un-hydrolyzed fecal mutagenicity by the inhibitor diet (Fig. 2, Table 3) is consistent with the binding of HCAs to CHL or to the cell walls of lactobacilli. CHL forms molecular complexes with planar mutagens, such as aflatoxin and HCAs, and it reduces the mutagenicity of these compounds in *Salmonella* and the uptake of these compounds in trout and rats [9]. Studies in rats suggest a combined effect of reduced mutagen uptake and increased conjugation by Phase II enzymes [30], [31].

HCAs can bind to the cell walls of lactobacilli *in vitro*, decreasing mutagenesis [23]. Lactobacilli also exhibit protective effects in rodent and human studies, with possible mechanisms including antioxidant activity as well as effects on cell proliferation and apoptosis [8]. As noted earlier, the greater fiber content of our inhibitor diet may account for some of the altered urinary and fecal mutagenicity, as well as reduced DNA damage in colorectal cells, produced by the inhibitor diet.

### DNA damage in WBCs and rectal cells

Vegetable juices reduced DNA damage in lymphocytes in some human dietary intervention studies [32]; however, we observed no effects of diet on DNA damage in WBCs, although our diets did not involve vegetable juices. In contrast, we found that diet altered DNA damage levels in rectal cells (Fig. 2E–F). Kiss et al. [33] showed that a fried-meat diet increased DNA damage in exfoliated colorectal mucosal cells compared to a vegetarian diet, particularly among subjects with *GSTM1-null* or *NAT2-rapid* genotypes. In rats, diets containing cooked beef or chicken increased DNA single- or double-strand breaks in colonocytes, but dietary fiber (high-amylose maize starch) inhibited such damage [34]. Other animal studies demonstrated the inhibition of HCA- or fried meat-induced DNA damage by lactobacilli [8] and by cruciferous vegetables and their constituents [26]. Given that both CHL and compounds in cruciferous vegetables induced apoptosis *in vitro* [15], [35], we cannot rule out apoptosis as a mechanism for decreased DNA damage with the inhibitor diet. To our knowledge, this study is the first in humans to demonstrate that dietary factors can protect rectal cells from DNA damage induced by mutagens, such as those in fried meat.

### Conclusions

Our study is limited by the small number of subjects (*n* = 16). In addition, we did not monitor changes in blood or urine levels of isothiocyanates or other components of cruciferous vegetables, CHL or chlorins, HCA-glucuronides in urine, or whether diets with lactobacilli altered the flora in fecal samples. The study design, *i.e.*, testing the effects of the three putative inhibitors together rather than testing each inhibitor separately, prevents conclusions about the relative importance of the three inhibitors and about their mechanism of DNA-damage inhibition. For example, we could not evaluate the possible interaction between the putative inhibitors, assessing whether their protective mechanisms may have been synergistic or antagonistic with each other. Future studies are warranted to study the protective mechanisms of each dietary inhibitor, cruciferous vegetables, chlorophyllin, and yogurt alone against HCA-induced genotoxicity. Although our study was not designed to examine differences between women and men in the responses that we measured, a study designed to confirm whether or not such differences exist is needed. Given the few subjects and short-term dietary regimens, our results must be interpreted with caution. Nevertheless, they indicate that meat cooked at high temperature increased mutagenicity in urine and feces and that consumption of yogurt, cruciferous vegetables, and CHL altered urinary and fecal mutagenicity and reduced colorectal cell DNA damage. Although increased urinary mutagenicity following consumption of highly fried meat is well established, our study is the first to concurrently measure both fecal mutagenicity and DNA damage in colon epithelium and to demonstrate that dietary antimutagens can alter these characteristics.

## Materials and Methods

### Study population and design

The protocol for this trial and supporting CONSORT checklist are available as supporting information; see Checklist S1 and Protocol S1. We conducted a randomized, controlled trial involving 16 healthy volunteers (8 men and 8 women) recruited by local advertisement from the Chapel Hill, NC area. Subjects were non-smokers, aged 18–45 (mean 29.5 y), had a body mass index <30, and were not currently taking prescription medications or antibiotics. Individuals were excluded if they consumed >2 alcoholic drinks/day, were vegetarians, or had a history of diabetes, goiter, colitis, a diagnosed thyroid condition, or occult bleeding.

Following a telephone interview to determine eligibility, we invited individuals for a second interview. At this time, we described the study, asked about dietary preferences, and administered a brief questionnaire on medical and diet history (including meat intake). Initial screening measurements for each subject included blood pressure, body temperature, and respiration rate. We recorded height and weight for diet calculations, instructed subjects to eat only the food provided by the CTRC, and prohibited use of aspirin, vitamins, and herbal supplements during the study.

The study was approved by the Institutional Review Boards of the School of Medicine at the University of North Carolina at Chapel Hill and the National Institute of Environmental Health Sciences, and by human studies approving officials at the U.S. Environmental Protection Agency. All subjects provided written informed consent.

This study had two arms, each a cross-over design (Fig. 1) that used 8 subjects (4 men and 4 women). For each arm, 2 men and 2 women were assigned randomly to a dietary sequence consisting of 2 weeks on one defined diet followed by two weeks on another; the remaining 4 subjects were assigned the same two diets in reverse order. In arm 1 (Fig. 1), one diet included beef and sausage cooked at low temperature (100°C); the other diet included the same meat cooked at high temperature (250°C). Both contained non-cruciferous vegetables. In arm 2 (Fig. 1), one diet included meat cooked at high-temperature along with non-cruciferous vegetables; the other diet included high-temperature meat along with the putative inhibitors (cruciferous vegetables, chlorophyllin tablets, and yogurt). Because of limited capacity at the Clinical Translational Research Center (CTRC) at the University of North Carolina at Chapel Hill, subjects were enrolled in groups of 4 for the 4-week protocols. At the beginning of each study period, subjects were chosen by the study coordinator for each arm and dietary sequence by random blind selection of lots containing subject names from the pool of subjects available to participate in that time period. Males and females were selected in separate random blind lots so that assignment to dietary regimes was balanced by gender for all arms and dietary sequences. Clinical personnel, samples, and laboratory personnel were all blinded to a subject's dietary regime.

All meals were prepared at the CTRC at the University of North Carolina at Chapel Hill. Subjects consumed breakfast and dinner at the CTRC and were provided with a packed lunch and snack.

### Meat preparation and diet

Preparation of meat fried at low (100°C for 10 min/side, followed by 20 min in an oven at 90°C to reduce moisture) and high (250°C for 11 min/side) temperature was based on a previous NCI study [18], with subjects consuming ∼3.8–4.2 g of cooked meat/kg body weight/day. For dietary sequences containing putative HCA inhibitors, subjects consumed meals containing 500 g of cruciferous vegetables/day (50% cooked and 50% raw), including green and red cabbage, broccoli, Brussels sprouts, and cauliflower; two or three 3.3-oz containers/day based on body weight of DanActive™, a probiotic cultured dairy drink containing 10^10^ CFUs of *L. casei* DN-114 001, *S. thermophilus*, and *L. bulgaricus* (Dannon, White Plains, NY); and 3 100-mg sodium copper chlorophyllin tablets/day (Derifil, Rystan Falls, NJ). All diets were designed for weight maintenance based on 35 kcal/kg body weight and contained 20% protein, 45% carbohydrate, and 35% fat.

Briefly, we purchased lean ground beef (15% fat, Harris Teeter, Mathews, NC) and sausage (Wampler's Farm Sausage, Inc. Lenoir City, TN) locally. For high-temperature meat diets, ground beef was cooked in 100-g patties at 250°C (482°F) on a commercial grill (Garland, New Port Richey, FL) for 11 min on each side for a total of 22 min then crumbled in an industrial mixer (Univex, Salem, NH), weighed and frozen. Sausage patties (85 g) were cooked as for ground beef and stored frozen as patties or crumbled meat. In a pilot study, we tested the mutagenicity of bacon (Hormel Griddlemaster, purchased locally) cooked at 250°C (482°F) for 11 min on a side. Because the mutagenicity and HCA levels for high-temperature bacon varied significantly from those of the high-temperature beef and sausage, we decided to use only beef and sausage cooked at low and high temperature for the main study. For low-temperature meat diets, beef and sausage were cooked at 100°C (212°F) for 10 min on each side and held in a 90°C oven for 20 min to reduce moisture [18]. Subjects consumed different amounts of meat, depending on body weight, ranging from 211 g/day for the lightest subject (50 kg) to 343 g/day for the heaviest subject (90 kg). The amount of high-temperature meat consumed by each subject was calculated to approximate the intake of HCAs/kg body weight in the previous NCI study [18].

We estimated amounts of inhibitors from previous studies; but these amounts were designed for an overall effect and not as a means of finding the optimal amounts for inhibition. We based the amount of cruciferous vegetables (500 g/day) on previous studies that showed effective reduction in urine mutagenicity and induction of GST isoforms [12]. The amount of CHL (300 mg/day) was a safe and effective dose based on a previous human-intervention study [36]. These inhibitors were consumed together with high-temperature beef and sausage. Without prior information on levels of lactobacilli needed for inhibition of HCAs, we arbitrarily gave subjects 2 or 3 3-oz. containers of DanActive based on body weight; subjects weighing over 65 kg received 3 containers/day.

Body weight was measured every weekday. To minimize weight changes, we adjusted calories for weight changes of more than 1 kg from baseline weight recorded at the start of the study. We designed three versions of the menu for each 2-week period to provide variety to the diet and administered them on a 3-day cycle in both phases of the study. Nutrient values of the foods used were based on the USDA Standard Reference nutrient database (Number 16) and calculated using commercial software (ProNutra, Version 3.1.0.13, Viocare Princeton, NJ).

### Meat analysis

Organic extracts of fried beef and sausage were prepared and analyzed for mutagenicity and for the following five HCAs as described previously [37], [38]: MeIQx (2-amino-3,8-dimethylimidazo[4,5-*f*]quinoxaline), 4,8-DiMeIQx (2-amino-3,4,8-trimethylimidazo[4,5-*f*]quinoxaline), 7,8-DiMeIQx (2-amino-3,7,8-trimethylimidazo[4,5-*f*]quinoxaline), PhIP (2-amino-1-methyl-6-phenylimidazo[4,5-*b*]pyridine), and IFP (2-amino-(1,6-dimethylfuro[3,2-e]imidazo[4,5-*b*])pyridine). Limits of detection were ∼1 ng/g.

### Collection of blood, rectal biopsies, urine, and stool

After consuming a dietary regimen for a full 7 days, subjects reported each week of the 4-week study to the CTRC for a blood draw and rectal biopsy. Subjects provided first-morning void urine samples each day throughout the study except on Saturdays and Sundays, and stool samples in the second and fourth weeks of the study.

Whole blood, collected in ACD (acid dextrose citrate) Vacutainer® tubes (Becton Dickinson, Franklin Lakes, NJ), was processed within 2 h of the blood draw for use in the comet assay. Leukocytes were separated from whole blood using ACCUSPIN® System-HISTOPAQUE®-1077 tubes (Sigma-Aldrich, St. Louis, MO) according to the manufacturer's instructions.

We obtained rectal biopsies using a disposable, flexible biopsy forceps (Boston Scientific, Natick, MA) mounted on a semi-rigid rod and inserted through a short (25 cm), rigid, disposable sigmoidoscope. The obdurator was removed, and the biopsy forceps were inserted to a depth of 10 cm. We obtained at least 4 pinch biopsies from each subject each week during the study. Biopsies were transferred from the forceps onto bibulous paper and stored in sterile 13-ml tubes (Becton Dickinson, Franklin Lakes, NJ) containing Hank's Buffered Saline Solution (HBSS, Gibco, Carlsbad, CA) with 0.3% bovine serum albumin (Gemini Bio-Products, Sacramento, CA) and 1% penicillin (100 U/ml) and streptomycin (0.1 mg/ml, Gibco). Samples were processed for use in the comet assay within 2 h of the biopsy procedure.

We isolated epithelial cells from rectal biopsies using a method described previously [39]. The 4 pinch biopsies from each subject were pooled, minced with fine scissors, and incubated with 6 mg of proteinase K (Invitrogen, Carlsbad, CA) and 3 mg of collagenase (Roche, Indianapolis, IN) in 3 ml HBSS in 15-ml tubes for 30 min in a shaker water bath at 37°C. Fresh HBSS was added to the tube to a volume of 12 ml, and the samples were centrifuged at 139× *g* for 6 min. Pellets were re-suspended in 200 µl HBSS for the comet assay and viability testing.

Urine was first stored either on ice or at 4°C until received that morning in the laboratory, at which time the samples were stored at −80°C until they were processed for mutagenicity testing. We determined the mutagenicity of organic extracts of samples collected each week (*i.e.*, days 7, 14, 21, and 28) after subjects consumed a particular dietary regimen for a full 7 days. In addition, subjects were asked to collect feces on days 14 and 28 of the study. We recorded wet weights of the fecal samples, and stored the samples at −80°C until processed for mutagenicity testing.

We thawed, lyophilized, and weighed fecal samples, and then extracted 1-g portions. For acid hydrolysis, 10 ml of 6-N HCl were added to 1 g of lyophilized feces, the sample was mixed thoroughly and then incubated for 6 h at 70°C. The pH was adjusted to 7.0 to 8.0, and 1 g of sodium carbonate was added as a buffer. Both hydrolyzed and un-hydrolyzed samples were extracted twice with dichloromethane (Burdick & Jackson, Morristown, NJ, pesticide grade, 99.9% minimum), filtered through glass wool to remove solid particles, concentrated to 10 ml by Turbovap®(Zymark Turbovap® II, Caliper Life Sciences, Hopkinton, MA), and stored at 4°C.

### Comet assay

We evaluated DNA damage in white blood cells (WBCs) and epithelial cells from rectal biopsies using a modified version of the comet assay described previously [39]. Rectal cells were incubated for 30 min with proteinase K and collagenase. WBCs (∼10,000)) from blinded samples in 10 µl phosphate-buffered saline (PBS, Gibco, Carlsbad, CA) or rectal biopsy cells (∼50,000) in 10 µl Hank's Buffered Saline Solution (HBSS, Gibco, Carlsbad, CA) were suspended in 100 µl of 1% (w/v) low-melting point agarose in PBS, pH 7.4 at 37°C, and pipetted immediately onto slides pre-coated with 1% (w/v) normal-melting point agarose. After a second layer of 100 µl of low-melting point agarose was pipetted on top of the previous layer, slides were allowed to cool for ∼10 min at 4°C and immersed in lysis solution (2.5-M NaCl, 100-mM Na_2_EDTA, 10-mM Tris, 1% (v/v) Triton-X 100, and NaOH at pH 13.0) at 4°C overnight. Following cell lysis, slides were neutralized for 5 min in 0.4-M Tris-HCl, pH 7.5 and then placed in a horizontal electrophoresis tank containing 0.3-M NaOH and 1-mM Na_2_EDTA, pH 13 to unwind the DNA for 40 min followed by electrophoresis at a constant voltage of 25 V for 40 min. We used these relatively long unwinding and electrophoresis times to detect the basal levels of damage associated with the different diets and to increase our ability to detect any protective effects of the inhibitor diet. Slides were washed 3 times for 5 min each with 0.4-M Tris-HCl, pH 7.5, placed in cold EtOH, dried, and stained with 20 µg/ml of ethidium bromide. We scored 50 comets/slide and 4 slides/sample, for a total of 200 cells/sample, using Komet 5.0 software (Kinetic Imaging Ltd., Liverpool, UK). We performed all experiments in low light to reduce background UV induction of DNA damage. We scored and expressed the results as Tail Moment (TM). Viability of leukocytes and rectal biopsy cells was determined as described [40] and was >80%.

### Mutagenicity assays

We evaluated fried meat extracts for mutagenicity in the *Salmonella* plate-incorporation assay [41] in the frameshift strain TA98 [*hisD3052 chl*-*1008* (*bio uvrB gal*) *rfa*-*1004* pKM101^+^ Fels-1^+^ Fels-2^+^ Gifsy-1^+^ Gifsy-2^+^] and two of its derivatives, YG1024 and YG1041. Strain YG1024 expresses acetyltransferase, which activates HCAs to mutagens [42], and YG1041 expresses both acetyltransferase and nitroreductase, which activates nitroarenes to mutagens [43]. Urine and stool extracts were tested only in YG1024. All extracts were tested in the presence of Arochlor-induced rat liver S-9 (Moltox, Boone, NC) at 2 mg of S9 protein/plate in one or two plates per dose, depending on the sample availability. Meat extracts were tested at a dose range of 0.06 to 1.25 g-eq/plate.

Organic extracts of urine were prepared in a manner similar that described previously [12], [17]. Briefly, 25–30 ml of urine were hydrolyzed in 6-N HCl for 6 h at 70°C. The hydrolysate and a similar portion of unhydrolyzed urine were extracted by C18/methanol, the extract was solvent-exchanged into DMSO at 150×, and the concentrate was frozen until tested for mutagenicity at 0.3 to 6.0 ml-eq/plate. We used acid hydrolysis because, as noted in our earlier studies [12], [17], acid hydrolysis is a highly effective method to deconjugate HCAs in urine. Doses were converted from ml-eq/plate to µmoles of creatinine/plate as described in the Supporting information (File S1, Table S5).

Fecal samples were lyophilized and weighed, a portion was hydrolyzed for 6 h at 70°C in 6-N HCl, the pH was adjusted to 7.0 to 8.0, and the sample was extracted twice with dichloromethane. The percent extractable organic matter (EOM) was determined by standard methods [44], and extracts were tested for mutagenicity at 40 to 400 µg of EOM/plate. In order to assess the mutagenic activity of the feces in a manner similar to that of the urine, we also used acid hydrolysis with the feces. Doses were converted from µg of EOM/plate to mg of lyophilized feces/plate as described in the Supporting information (File S1, Table S9).

### Statistical analyses

#### Levels of HCAs in meat

We computed means, standard deviations, and 95% confidence limits for HCA levels using data from 4 independent preparations each of beef and sausage cooked at high temperature.

#### Estimation of mutagenic potency: general considerations

The data from a single plate-incorporation assay consist of counts of mutant colonies from one or more plates at each in a series of increasing concentrations (doses) of the extract under test. The underlying theory holds that the mutagenic potency of the extract is the slope of the linear portion of the resulting dose-response relationship. Consequently, because toxicity may cause the dose-response trajectory to flatten or turn down at high doses, we followed toxicologic practice in allowing only doses deemed within the linear range to contribute to estimation [41]. In the Supporting Information (File S1), we provide dose-specific average counts for every assay that we conducted.

Let 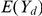 denote the expected (mean) number of mutant colonies at dose *d*. For estimating mutagenic potency based on a single assay, we modeled the expected counts as a linear function of dose, namely, 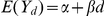 where 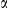, the expected count at dose 0, reflects the background mutant yield in the *Salmonella* strain used in the assay and 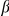, the slope, is the mutagenic potency. We regarded plate counts as Poisson distributed but allowed for over-dispersion among plates within an assay. This model is a generalized linear model (GLM) with a Poisson error distribution, the identity link function, and over-dispersion parameter [45]. We fit such GLMs using PROC GENMOD in SAS version 9.1 (SAS Institute, Cary, NC).

For estimating mutagenic potency when multiple specimens or multiple assays per specimen contributed to a common average mutagenic potency, we used closely related but more elaborate models that reflected sources of variation in the data and assumptions about assay properties. If we assayed multiple specimens (from the same or different treatments) on the same day, their assays all shared the same dose 0 plate(s), reflecting that the background mutant yield is regarded as the same for all specimens on a given day; however, separate dose 0 plates were run on different days, reflecting that uncontrolled day-to- day fluctuations in the laboratory environment could induce minor day-to-day disturbances in the background mutant yield. Similarly, we expected mutagenic potency to vary slightly from assay to assay if, for example, a given specimen was assayed on multiple days or if different specimens representing a given treatment were assayed on the same day. To reflect such sources of extra-Poisson variation, we used generalized linear mixed models (GLMMs) [46]. As a simple example, consider that we assayed a separate preparation of beef cooked at high temperature on each of 3 days in strain YG1024. Our statistical analysis was based on the following model:
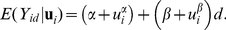
(1)Here, 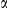 and 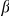 have the same interpretations as earlier, 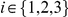 indexes day (or, equivalently here, preparation or assay), 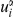 and 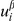 represent disturbances on day *i* to the intercept and to the mutagenic potency, respectively, attributable to random day-to-day variation among assays, and 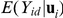 represents the expected number of mutant colonies at dose 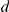 on day *i* conditional on the values of the disturbances. As before, we regarded plate counts as Poisson distributed; in addition, the disturbances 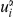 and 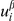 (termed ‘random effects’) were modeled as independent mean-zero Gaussian random variables, each with its own variance. We modeled over-dispersion through a residual error variance. We fit such GLMMs using the SAS GLIMMIX macro [47].

We applied GLMMs more elaborate than model (1) as the experiment's design required. If an analysis involved estimation of mutagenic potencies for more than one treatment, model (1) was extended to include additional slopes (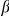-terms) to estimate mutagenic potency for each treatment. In addition, analyses for more complicated designs, like our crossover designs, involved additional possible random disturbances to each slope, that is, additional terms analogous to 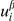 in model (1). For crossover designs, we also considered carryover [48] from the diet given in one period onto the diet given in the following period to influence slopes. Because specimen-to-specimen or assay-to-assay variation in mutagenic potency tends to be larger for treatments with higher mutagenic potency, our models incorporated heterogeneity of variances across treatments for certain random effects. To choose a parsimonious specification for modeling the random effects in a GLMM, we used BIC [49], a widely used model-selection criterion. Also, we checked that the estimated random effects obeyed the Gaussian distribution assumption. In the following subsections, we indicate when we used additional slopes, what random effects were modeled for slopes, and whether the models involved heterogeneous variances. All our GLMMs, like model (1), modeled only day as having a random effect on the intercept.

#### Estimation of mutagenic potency: meat extracts

Our pilot study assayed three types of meat (bacon, beef, sausage) cooked at two temperatures (low, high) in three different strains of *Salmonella* (TA98, YG1024, YG1041). Each assay involved one or two plates (depending on specimen volume available) at each of 6 doses (g-eq/plate), including zero. We estimated the mean mutagenic potency and its standard error for each treatment combination of type, temperature, and strain by fitting a separate model for each treatment combination. Treatment combinations that involved a single meat preparation assayed once were analyzed using a GLM. Two treatment combinations (Table 1), however, involved three independent meat preparations, each assayed on a separate day, and were analyzed using the GLMM of model (1), which included a random effect of day on mutagenic potency.

During the feeding study, we used strain YG1024 to monitor the mutagenic potency of two types of meat (beef, sausage) cooked at high temperature, each type represented by 4 independent preparations. Assays were conducted on three separate days (one beef and one sausage preparation each on two days; two beef and two sausage preparations on the third). Each assay used one plate per dose level. On each day, we used a single zero-dose plate for both types of meat and, therefore, analyzed both types in a single statistical analysis with a GLMM. We modeled expected plate counts as two lines with a common intercept but separate slopes for beef and sausage. We included random effects on mutagenic potencies (meat-type-specific slopes) for days and for preparations within days.

#### Estimation of mutagenic potency: urine

We collected urine specimens from every subject in every period of the crossover design (Fig. 1) (though one specimen had sufficient volume for only an unhydrolyzed sub-sample) and conducted assays on 4 days for arm-1 samples and 4 days for arm-2 samples. On each day, one set of 3 or 6 zero dose plates was used for all assays. Each specimen, including both the hydrolyzed and unhydrolyzed sub-samples, was assayed on only one day. Each assay involved 4–5 non-zero doses (µmole creatinine) with one plate per dose. We analyzed data from each arm separately with the same method of analysis. We estimated mutagenic potencies and their standard errors for each diet with both hydrolyzed and unhydrolyzed extracts (4 potency estimates per arm); we also tested for differences in mutagenic potency between diets separately for hydrolyzed and for unhydrolyzed extracts.

For each arm, we analyzed data on diet and acid hydrolysis treatment together using a GLMM. We modeled the expected plate counts as 4 lines with a common intercept and 4 separate slopes, one for each combination of diet and hydrolysis treatment; we also allowed for possible carryover effects on slopes. The GLMM included random effects of subjects, periods, specimens, sub-samples on the slopes as well as residual variation. We accommodated variance heterogeneity by allowing the variances of random effects for specimens, sub-samples, and residual variation to depend on treatment combination. The estimated mutagenic potencies that we report are adjusted for carryover effects through the fitted model.

#### Estimation of mutagenic potency: feces

The crossover design for fecal specimens involved two periods (Fig. 1); we collected a specimen from every subject in each period. Each specimen was represented by a hydrolyzed and an un-hydrolyzed sub-sample. Plate-incorporation assays were conducted on 7 days for arm-1 and 3 days for arm-2 specimens. On each day, all specimens were represented by 2 or 3 zero-dose plates. Each specimen, including both sub-samples, was assayed on three distinct days (replicate assays). Each replicate assay involved 4–5 non-zero doses (mg lyophilized feces) with 1 or 2 plates per dose, depending on available specimen volume.

We analyzed each arm of the study separately using the same GLMM. As with urine, we modeled the expected plate counts as 4 lines with a common intercept and 4 separate slopes, one for each combination of diet and hydrolysis treatment; we also allowed for possible carryover effects on slopes. The GLMM for feces differed from that for urine in accommodating replicate assays of the same sub-samples. It included random effects of subjects, periods, specimens, and replicate assays on the slopes as well residual variation and allowed the variances of random effects for specimens, assays, and residual variation to depend on treatment combination. The estimated mutagenic potencies that we report are adjusted for carryover effects through the fitted model.

#### Estimation of DNA damage in leukocytes and colon epithelium cells

We had both leukocyte (WBC) samples and rectal epithelium biopsy specimens from every subject each week during each arm of our crossover study (Fig. 1). All specimens (WBCs and rectal cells) were evaluated by the comet assay on the day that they were collected. For each specimen, we computed median TM values from the comet assay across 50 cells per slide and averaged these medians across 4 slides; thus, our DNA-damage measure represented a typical value among 200 cells per specimen. We analyzed DNA damage in each arm and in leukocytes and rectal epithelium separately, four analyses in all. We employed mixed-model analysis-of-variance techniques appropriate for our crossover design [48] using PROC MIXED™ in SAS 9.1. In each arm, after verifying that carryover effects were unimportant (p>0.25), we estimated the mean response for each diet using a model that included random effects of subject, period, and residual error. We report model-based estimates and confidence limits for mean TM for each diet.

## Supporting Information

File S1 Data from mutagenicity and comet assay experiments.
(DOC)

Checklist S1 CONSORT Checklist.
(DOC)

Protocol S1 Trial Protocol.
(DOC)
