# Supplementary material for: Inhibition of Fried Meat-Induced Colorectal DNA Damage and Altered Systemic Genotoxicity in Humans by Crucifera, Chlorophyllin, and Yogurt
Source: PLoS One. 2011 Apr 25;6(4):e18707. doi: 10.1371/journal.pone.0018707 (PMC3081825; doi:10.1371/journal.pone.0018707)
Supplement: File S1 — Data from mutagenicity and comet assay experiments. (DOC) [file pone.0018707.s001.doc]

**Supplementary Material**

Table S1 - Meat Mutagenicity

| Mutagenicity of meat extracts in *S. typhimurium* TA98 (pilot study) | | | | | | | | |
| --- | --- | --- | --- | --- | --- | --- | --- | --- |
|  |  |  |  |  |  |  |  |  |
|  | Beef | LT^a^ |  | Beef | HT^b^ |  | Beef | HT^b^ |
|  | Exp 1 |  |  | Exp 1 |  |  | Exp 2 |  |
|  | g-eq./plate |  |  | g-eq./plate |  |  | g-eq./plate |  |
|  | 0 | **28^c^** |  | 0 | **36** |  | 0 | **36** |
|  | 0.003 | **22** |  | 0.003 | **46** |  | 0.003 | **49** |
|  | 0.006 | **26** |  | 0.005 | **54** |  | 0.005 | **67** |
|  | 0.030 | **31** |  | 0.026 | **80** |  | 0.026 | **95** |
|  | 0.06 | **32** |  | 0.051 | **120** |  | 0.051 | **137** |
|  | 0.152 | **28** |  | 0.128 | **256** |  | 0.128 | **222** |
|  | 0.303 | **20** |  | 0.252 | **367** |  | 0.252 | **323** |
|  | 0.606 | **36** |  | 0.512 | **605** |  | 0.512 | **515** |
|  |  |  |  |  |  |  |  |  |
|  | Beef | HT^b^ |  | Sausage | HT^b^ |  | Bacon | HT^b^ |
|  | Exp 3 |  |  | Exp 1 |  |  | Exp 1 |  |
|  | g-eq./plate |  |  | g-eq./plate |  |  | g-eq./plate |  |
|  | 0 | **36** |  | 0 | **28** |  | 0 | **28** |
|  | 0.003 | **48** |  | 0.003 | **34** |  | 0.001 | **21** |
|  | 0.005 | **60** |  | 0.006 | **39** |  | 0.002 | **38** |
|  | 0.027 | **88** |  | 0.030 | **43** |  | 0.011 | **75** |
|  | 0.054 | **129** |  | 0.059 | **82** |  | 0.022 | **100** |
|  | 0.135 | **239** |  | 0.148 | **169** |  | 0.055 | **234** |
|  | 0.269 | **345** |  | 0.295 | 218 |  | 0.110 | 246 |
|  | 0.538 | **560** |  | 0.59 | 295 |  | 0.219 | 374 |

^a^Beef patties were cooked at 100°C for 10 min on each side.

^b^Beef, sausage, and bacon were cooked at 250°C for 11 min on each side.

^c^Counts designated as within the linear portion of the dose-response curve are in bold. Counts for control (0 g-eq./plate) plates are the average of 3 separate plates. All other concentrations were tested in single plates.

Table S1 - continued

| Mutagenicity of meat extracts in *S. typhimurium* YG1024 (pilot study) | | | | | | | | |
| --- | --- | --- | --- | --- | --- | --- | --- | --- |
|  |  |  |  |  |  |  |  |  |
|  | Beef | LT^a^ |  | Beef | HT^b^ |  | Beef | HT^b^ |
|  | Exp 1 ^d^ |  |  | Exp 1^d^ |  |  | Exp 2 ^d^ |  |
|  | g-eq./plate |  |  | g-eq./plate |  |  | g-eq./plate |  |
|  | 0 | **37** ^c^ |  | 0 | **79** |  | 0 | **79** |
|  | 0.003 | **20** |  | 0.003 | **86** |  | 0.003 | **114** |
|  | 0.006 | **33** |  | 0.005 | **151** |  | 0.005 | **134** |
|  | 0.030 | **48** |  | 0.026 | **428** |  | 0.026 | **374** |
|  | 0.06 | **42** |  | 0.051 | **842** |  | 0.051 | **557** |
|  | 0.152 | **52** |  | 0.128 | **1451** |  | 0.128 | **1148** |
|  | 0.303 | **47** |  | 0.252 | 2061 |  | 0.252 | 1564 |
|  | 0.606 | **30** |  | 0.512 | 2764 |  | 0.512 | 1848 |
|  |  |  |  |  |  |  |  |  |
|  | Beef | HT^b^ |  | Sausage | HT^b^ |  | Bacon | HT^b^ |
|  | Exp 3 ^d^ |  |  | Exp 1 ^d^ |  |  | Exp 1^d^ |  |
|  | g-eq./plate |  |  | g-eq./plate |  |  | g-eq./plate |  |
|  | 0 | **79** |  | Beef | **37** |  | 0 | **37** |
|  | 0.003 | **100** |  | 0.003 | **63** |  | 0.001 | **92** |
|  | 0.005 | **143** |  | 0.006 | **96** |  | 0.002 | **123** |
|  | 0.027 | **401** |  | 0.030 | **329** |  | 0.011 | **522** |
|  | 0.054 | **700** |  | 0.059 | **459** |  | 0.022 | **664** |
|  | 0.135 | **1300** |  | 0.148 | **1003** |  | 0.055 | **1375** |
|  | 0.269 | 1813 |  | 0.295 | 1475 |  | 0.110 | 1771 |
|  | 0.538 | 2306 |  | 0.59 | 1680 |  | 0.219 | 2446 |
|  |  |  |  |  |  |  |  |  |

^a^Beef patties were cooked at 100°C for 10 min on each side.

^b^Beef, sausage, and bacon were cooked at 250°C for 11 min on each side.

^c^Counts designated as within the linear portion of the dose-response curve are in bold. Counts for control (0 g-eq./plate) plates are the average of 3 separate plates. All other concentrations were tested in single plates.

Table S1 - continued

| Mutagenicity of meat extracts in *S. typhimurium*YG1041 (pilot study) | | | | | | | | |
| --- | --- | --- | --- | --- | --- | --- | --- | --- |
|  |  |  |  |  |  |  |  |  |
|  | Beef | LT^a^ |  | Beef | HT^b^ |  |  |  |
|  | Exp 1 |  |  | Exp 1 |  |  |  |  |
|  | g-eq./plate |  |  | g-eq./plate |  |  |  |  |
|  | 0 | **62**^c^ |  | 0 | **61** |  |  |  |
|  | 0.003 | **59** |  | 0.003 | **115^d^** |  |  |  |
|  | 0.006 | **69** |  | 0.005 | **99^d^** |  |  |  |
|  | 0.030 | **64** |  | 0.026 | **337^d^** |  |  |  |
|  | 0.06 | **67** |  | 0.057 | **555^d^** |  |  |  |
|  | 0.152 | **75** |  | 0.128 | **1156^d^** |  |  |  |
|  | 0.303 | **65** |  |  |  |  |  |  |
|  | 0.606 | **69** |  |  |  |  |  |  |
|  |  |  |  |  |  |  |  |  |
|  | Sausage | HT^b^ |  | Bacon | HT^b^ |  |  |  |
|  | Exp 1 |  |  | Exp 1 |  |  |  |  |
|  | g-eq./plate |  |  | g-eq./plate |  |  |  |  |
|  | 0 | **62** |  | 0 | **62** |  |  |  |
|  | 0.003 | **82** |  | 0.001 | **95** |  |  |  |
|  | 0.006 | **84** |  | 0.002 | **125** |  |  |  |
|  | 0.030 | **256** |  | 0.011 | **348** |  |  |  |
|  | 0.059 | **349** |  | 0.022 | **432** |  |  |  |
|  | 0.148 | **802** |  | 0.055 | **967** |  |  |  |
|  | 0.295 | **920** |  | 0.110 | 1338 |  |  |  |
|  | 0.59 | **1438** |  | 0.219 | 1572 |  |  |  |

^a^Beef patties were cooked at 100°C for 10 min on each side.

^b^Beef ,sausage and bacon were cooked at 250°C for 11 min on each side.

^c^Counts designated as within the linear portion of the dose-response curve are in bold. Counts for control (0 g-eq./plate) plates are the average of 3 separate plates. All other concentrations were tested in single plates except where noted.

^d^Counts are the average of two plates.

Table S1 - continued

| Mutagenicity of meat extracts in *S. typhimurium*YG1024 (feeding study) | | | | | | | | |
| --- | --- | --- | --- | --- | --- | --- | --- | --- |
|  |  |  |  |  |  |  |  |  |
|  | Beef | HT^b^ |  | Beef | HT^b^ |  | Beef | HT^b^ |
|  | Exp 1 |  |  | Exp 2 |  |  | Exp 3 |  |
|  | g-eq./plate |  |  | g-eq./plate |  |  | g-eq./plate |  |
|  | 0 | **52** |  | 0 | **52** |  | 0 | **48** |
|  | 0.003 | **45** |  | 0.003 | **63** |  | 0.003 | **67** |
|  | 0.006 | **72** |  | 0.006 | **62** |  | 0.006 | **103** |
|  | 0.015 | **118** |  | 0.015 | **159** |  | 0.03 | **323** |
|  | 0.03 | **418** |  | 0.03 | **301** |  | 0.06 | **468** |
|  | 0.06 | **485** |  | 0.06 | **601** |  | 0.15 | **1140** |
|  |  |  |  |  |  |  | 0.30 | 1301 |
|  |  |  |  |  |  |  | 0.60 | 1564 |
|  |  |  |  |  |  |  |  |  |
|  | Beef | HT^b^ |  | Sausage | HT^b^ |  | Sausage | HT^b^ |
|  | Exp 4 |  |  | Exp 1 |  |  | Exp2 |  |
|  | g-eq./plate |  |  | g-eq./plate |  |  | g-eq./plate |  |
|  | 0 | **45^d^** |  | 0 | **52** |  | 0 | **52** |
|  | 0.003 | **98^d^** |  | 0.003 | **63** |  | 0.003 | **58** |
|  | 0.006 | **164^d^** |  | 0.006 | **56** |  | 0.006 | **90** |
|  | 0.03 | **669^d^** |  | 0.015 | **136** |  | 0.015 | **155** |
|  | 0.06 | **1153^d^** |  | 0.03 | **301** |  | 0.03 | **360** |
|  | 0.15 | 2046^d^ |  | 0.06 | **437** |  | 0.06 | **583** |
|  | 0.30 | 2751^d^ |  |  |  |  |  |  |
|  | 0.60 | 3223^d^ |  |  |  |  |  |  |
|  |  |  |  |  |  |  |  |  |
|  | Sausage | HT^b^ |  | Sausage | HT^b^ |  |  |  |
|  | Exp3 |  |  | Exp 4 |  |  |  |  |
|  | g-eq./plate |  |  | g-eq./plate |  |  |  |  |
|  | 0 | **48** |  | 0 | **45** ^c^ |  |  |  |
|  | 0.003 | **65** |  | 0.003 | **113^d^** |  |  |  |
|  | 0.006 | **90** |  | 0.006 | **182 ^d^** |  |  |  |
|  | 0.03 | **245** |  | 0.03 | **593 ^d^** |  |  |  |
|  | 0.06 | **375** |  | 0.06 | **1024 ^d^** |  |  |  |
|  | 0.15 | **732** |  | 0.15 | 1911 **^d^** |  |  |  |
|  | 0.30 | 890 |  | 0.30 | 2685 **^d^** |  |  |  |
|  | 0.60 | 1085 |  | 0.60 | 3207 **^d^** |  |  |  |

^a^Beef patties were cooked at 100°C for 10 min on each side.

^b^Beef ,sausage and bacon were cooked at 250°C for 11 min on each side.

^c^Counts designated as within the linear portion of the dose-response curve are in bold. Counts for control (0 ml-eq./plate) plates are the average of 3 separate plates. All other concentrations were tested in single plates except where noted.

^d^Counts are the average of two plates.

Table S2

| Urine mutagenicity after consumption of the meal containing meat fried at low temperature^a^ | | | | | | | | | | | |
| --- | --- | --- | --- | --- | --- | --- | --- | --- | --- | --- | --- |
| Sample ID: | #1^b^ | week 1 |  | #1 | week 2 |  | #2 | week 3 |  | #2 | week 4 |
| ml-eq./plate | U | H |  | U | H |  | U | H |  | U | H |
| 0 | **48**^d^ | **48** |  | **48** | **48** |  | **48** | **48** |  | **48** | **48** |
| 0.3 | **50** | **71** |  | **53** | **56** |  | **52** | **290** |  | **58** | **94** |
| 0.75 | **53** | **86** |  | **56** | **74** |  | **55** | **447** |  | **63** | **99** |
| 1.5 | **62** | **96** |  | **61** | 56 |  | **77** | **561** |  | **69** | 96 |
| 3 | **84** | 37 |  | **87** | 32 |  | **90** | 287 |  | **103** | 89 |
| 6 | **136** | 15 |  | **91** | 39 |  | **99** | 508 |  | **128** | 57 |
|  |  |  |  |  |  |  |  |  |  |  |  |
|  | #3 | week 1 |  | #3 | week 2 |  | #4 | week 3 |  | #4 | week 4 |
| ml-eq./plate | U | H |  | U | H |  | U | H |  | U | H |
| 0 | **48** | **48** |  | **48** | no data |  | **48** | **48** |  | **48** | **48** |
| 0.3 | **48** | **88** |  | **55** |  |  | **60** | **71** |  | **47** | **70** |
| 0.75 | **41** | **131** |  | **43** |  |  | **60** | **88** |  | **65** | **73** |
| 1.5 | **44** | **185** |  | **60** |  |  | **55** | **107** |  | **55** | **101** |
| 3 | **65** | **352** |  | **67** |  |  | **55** | **250** |  | **83** | **165** |
| 6 | **98** | 190 |  | **66** |  |  | **93** | 315 |  | **80** | **205** |
|  |  |  |  |  |  |  |  |  |  |  |  |
|  | #5 | week 1 |  | #5 | week 2 |  | #6 | week 1 |  | #6 | week 2 |
| ml-eq./plate | U | H |  | U | H |  | U | H |  | U | H |
| 0 | **62^c^** | **62** |  | **58** | **50** |  | **58** | **58** |  | **62** | **62** |
| 0.03 |  |  |  |  |  |  |  | **61** |  |  |  |
| 0.075 |  |  |  |  |  |  |  | **72** |  |  |  |
| 0.15 |  |  |  |  |  |  |  | **70** |  |  |  |
| 0.3 | **86** | **91** |  | **74** | **65** |  | **77** | **107** |  | **68** | **94** |
| 0.75 | **104** | **104** |  | **90** | **90** |  | **132** | 105 |  | **109** | **126** |
| 1.5 | **134** | 101 |  | **134** | 79 |  | **218** |  |  | **266** | 143 |
| 3 | **230** | 98 |  | **227** |  |  | **368** |  |  | **440** | 126 |
| 6 | **349** | 68 |  | **415** |  |  | **718** |  |  | **718** | 67 |
|  |  |  |  |  |  |  |  |  |  |  |  |
|  | #7^b^ | week 3 |  | #7 | week 4 |  | #8 | week 3 |  | #8 | week 4 |
| ml-eq./plate | U | H |  | U | H |  | U | H |  | U | H |
| 0 | **62** | **62** |  | **62** | **62** |  | **62** | **62** |  | **58** | **58** |
| 0.03 |  |  |  |  |  |  |  |  |  | **54** | **56** |
| 0.075 |  |  |  |  |  |  |  |  |  | **63** | **61** |
| 0.15 |  |  |  |  |  |  |  |  |  | **64** | **84** |
| 0.3 | **85** | **63** |  | **67** | **87** |  | **66** | **87** |  | **49** | **110** |
| 0.75 | **82** | **102** |  | **69** | **130** |  | **57** | **73** |  | **65** | **133** |
| 1.5 | **78** | **186** |  | **93** | **133** |  | **84** | **107** |  |  |  |
| 3 | **134** | **314** |  | **144** | 90 |  | **98** | 36 |  |  |  |
| 6 | **182** | 222 |  | **178** | 77 |  | **119** | 39 |  |  |  |

^a^Beef and sausage patties were cooked at 100°C for 10 min on each side.

^b^Indicates subject number. Subjects consumed diets containing meat cooked at either low or high temperature for 2-week periods in a cross-over design.

^c^Urine was tested at lower concentrations when higher concentrations were toxic. Counts for control (0 ml-eq./plate) plates are the average of 3 separate plates. All other concentrations were tested in single plates.

^d^Counts designated as within the linear portion of the dose-response curve are in bold type. Assays with the same zero dose mean count were conducted on the same day.U = unhydrolyzed extracts H = acid-hydroylzed extracts

Table S3

| Urine Mutagenicity after consumption of the meal containing meat fried at high temperature^a^ | | | | | | | | | | | |
| --- | --- | --- | --- | --- | --- | --- | --- | --- | --- | --- | --- |
| Sample ID: | #1^b^ | week 3 |  | #1 | week 4 |  | #2 | week 1 |  | #2 | week 2 |
| ml-eq./plate | U | H |  | U | H |  | U | H |  | U | H |
| 0 | **48** ^d^ | **48** |  | **48** | **48** |  | **48** | **48** |  | **48** | **48** |
| 0.3 | **70** | **120** |  | **90** | **106** |  | **70** | **269** |  | **72** | **1315** |
| 0.75 | **73** | **155** |  | **109** | **101** |  | **102** | **350** |  | **90** | **1586** |
| 1.5 | **79** | **218** |  | **165** | 64 |  | **142** | **480** |  | **150** | **2445** |
| 3 | **187** | 245 |  | **281** | 29 |  | **400** | 427 |  | **372** | 2103 |
| 6 | **290** | 146 |  | 376 | 25 |  | **518** | 147 |  | 415 | 1516 |
|  |  |  |  |  |  |  |  |  |  |  |  |
| Sample ID: | #3 | week 3 |  | #3 | week 4 |  | #4 | week 1 |  | #4 | week 2 |
| ml-eq./plate | U | H |  | U | H |  | U | H |  | U | H |
| 0 | **48** | **48** |  | **48** | **48** |  | **48** | **48** |  | **48** | **48** |
| 0.3 | **85** | **270** |  | **110** | **265** |  | **55** | **155** |  | **56** | **230** |
| 0.75 | **110** | **428** |  | **165** | **377** |  | **62** | **209** |  | **64** | **633** |
| 1.5 | **111** | 606 |  | **301** | 410 |  | **78** | 240 |  | **90** | 898 |
| 3 | **430** | 267 |  | **670** | 116 |  | **259** | 113 |  | **197** | 633 |
| 6 | **737** | 81 |  | **858** | 74 |  | 238 | 82 |  | **278** | 230 |
|  |  |  |  |  |  |  |  |  |  |  |  |
| Sample ID: | #5^b^ | week 3 |  | #5 | week 4 |  | #6 | week 3 |  | #6 | week 4 |
| ml-eq./plate | U | H |  | U | H |  | U | H |  | U | H |
| 0 | **62** ^d^ | **50** |  | **62** | **62** |  | **58** | **58** |  | **62** | **62** |
| 0.03 |  |  |  |  |  |  | **80** | **55** |  |  |  |
| 0.075 |  |  |  |  |  |  | **78** | **137** |  |  |  |
| 0.15 |  | **65** |  |  |  |  | **176** | **186** |  |  |  |
| 0.3 | **31** | **81** |  | **118** | **381** |  | **200** | 237 |  | **44** | **492** |
| 0.75 | **30** | **102** |  | **148** | **449** |  |  | 155 |  | **41** | **620** |
| 1.5 | **107** | 126 |  | **219** | 410 |  |  |  |  | **391** | 452 |
| 3 | **155** |  |  | **558** | 145 |  |  |  |  | **999** | 97 |
| 6 | **237** |  |  | **804** | 75 |  |  |  |  | 885 | 70 |
|  |  |  |  |  |  |  |  |  |  |  |  |
| Sample ID: | #7 | week 1 |  | #7 | week 2 |  | #8 | week 1 |  | #8 | week 2 |
| ml-eq./plate | U | H |  | U | H |  | U | H |  | U | H |
| 0 | **62** | **62** |  | **62** | **62** |  | **62** | **58** |  | **62** | **58** |
| 0.03 |  |  |  |  |  |  |  | **64** |  |  | **61** |
| 0.075 |  |  |  |  |  |  |  | **102** |  |  | **97** |
| 0.15 |  |  |  |  |  |  |  | **131** |  |  | **64** |
| 0.3 | **50** | **323** |  | **86** | **312** |  | **68** | **209** |  | **61** | **127** |
| 0.75 | **58** | **604** |  | **77** | **599** |  | **88** | 216 |  | **66** | **156** |
| 1.5 | **76** | 820 |  | **84** | 570 |  | **70** |  |  | **67** |  |
| 3 | **84** | 729 |  | **137** | 463 |  | **88** |  |  | **80** |  |
| 6 | **99** | 239 |  | **156** |  |  | **133** |  |  | **114** |  |

Table S3 – continued

| Urine mutagenicity after consumption of the meal containing meat fried at high temperature^a^ | | | | | | | | | | | |
| --- | --- | --- | --- | --- | --- | --- | --- | --- | --- | --- | --- |
| Sample ID: | #9^b^ | week 1 |  | #9 | week 2 |  | #10 | week 1 |  | #10 | week 2 |
| ml-eq./plate | U | H |  | U | H |  | U | H |  | U | H |
| 0 | **62** ^d^ | **62** |  | **62** | **62** |  | **62** | **62** |  | **62** | **62** |
| 0.03 |  |  |  |  | **177** |  |  |  |  |  |  |
| 0.075 |  |  |  |  | **268** |  |  |  |  |  |  |
| 0.15 |  |  |  |  | 254 |  |  |  |  |  |  |
| 0.3 | **89** | **159** |  | **127** | 111 |  | **91** | **141** |  | **98** | **214** |
| 0.75 | **84** | **296** |  | **99** | 136 |  | **123** | **200** |  | **126** | **297** |
| 1.5 | **113** | 402 |  | **156** |  |  | **171** | 131 |  | **178** | 231 |
| 3 | **183** | 404 |  | **227** |  |  | **320** | 114 |  | **304** | 129 |
| 6 | **244** | 181 |  | **376** |  |  | **498** | 132 |  | **597** | 109 |
|  |  |  |  |  |  |  |  |  |  | |  |
| Sample ID: | #11 | week 3 |  | #11 | week 4 |  | #12 | week 3 |  | #12 | week 4 |
| ml-eq./plate | U | H |  | U | H |  | U | H |  | U | H |
| 0 | **62** | **62** |  | **62** | **62** |  | **62** | **62** |  | **62** | **62** |
| 0.3 | **106** | **295** |  | **130** | **331** |  | **101** | **226** |  | **88** | **199** |
| 0.75 | **205** | **299** |  | **204** | **372** |  | **115** | **222** |  | **110** | **223** |
| 1.5 | **330** | 122 |  | **291** | 314 |  | **186** | 113 |  | **185** | 125 |
| 3 | **742** | 101 |  | **741** | 106 |  | **415** | 74 |  | **404** | 94 |
| 6 | 806 | 109 |  | 914 | 97 |  | **540** | 51 |  | **510** | 65 |
| Sample ID: | #13 | week 1 |  | #13 | week 2 |  | #14 | week 1 |  | #14 | week 2 |
| ml-eq./plate | U | H |  | U | H |  | U | H |  | U | H |
| 0 | **64** ^c^ | **64** |  | **64** | **64** |  | **53** | **53** |  | **53** | **53** |
| 0.15 |  |  |  |  |  |  |  | **120** |  |  | **71** |
| 0.3 | **70** | **276** |  | **108** | **428** |  | **72** | **218** |  |  | **125** |
| 0.75 | **101** | **492** |  | **144** | **738** |  | **75** | **515** |  | **96** | **193** |
| 1.5 | **160** | 507 |  | **248** | 667 |  | **119** | **556** |  | **147** | **376** |
| 3 | **343** | 137 |  | **520** | 252 |  | **271** | **1636** |  | **390** | 394 |
| 6 | 285 | 79 |  | **756** | 63 |  | **381** |  |  | 479 |  |
|  |  |  |  |  |  |  |  |  |  |  |  |
| Sample ID: | #15^b^ | week 3 |  | #15 | week 4 |  | #16 | week 3 |  | #16 | week 4 |
| ml-eq./plate | U | H |  | U | H |  | U | H |  | U | H |
| 0 | **53** ^d^ | **53** |  | **53** | **53** |  | **64** | **64** |  | **64** | **64** |
| 0.075 |  |  |  |  | **136** |  |  |  |  |  |  |
| 0.15 |  | **110** |  |  | **176** |  |  |  |  |  |  |
| 0.3 | **100** | **307** |  | **80** | **265** |  | **97** | **248** |  | **83** | **235** |
| 0.75 | **165** | 399 |  | **154** | **388** |  | **103** | **330** |  | **111** | **257** |
| 1.5 |  | 422 |  |  | 350 |  | **218** | 226 |  | **143** | 187 |
| 3 |  |  |  |  |  |  | **472** | 79 |  | **299** | 106 |
| 6 | **870** |  |  | 570 |  |  | **552** | 52 |  | **421** | 421 |

^a^Beef and sausage patties were cooked at 250°C for 11 min on each side.

^b^Indicates subject number. Subjects consumed diets containing meat cooked at high temperature alone or with inhibitors for 2-week periods in a cross-over design.

^c^Urine was tested at lower concentrations when higher concentrations were toxic. Counts for control (0 ml-eq./plate) plates are the average of 3 separate plates. All other concentrations were tested in single plates.

^d^Counts designated as within the linear portion of the dose-response curve are in bold type.

U = unhydrolyzed extracts H = acid-hydroylzed extracts

Table S4

| Urine mutagenicity after consumption of the meal containing meat fried at high temperature^a^ together with dietary inhibitors^b^ | | | | | | | | | | | |
| --- | --- | --- | --- | --- | --- | --- | --- | --- | --- | --- | --- |
| Sample ID: | #9^c^ | week 3 |  | #9 | week 4 |  | #10 | week 3 |  | #10 | week 4 |
| ml-eq./plate | U | H |  | U | H |  | U | H |  | U | H |
| 0 | **62** ^c^ | **62^d^** |  | **62** | **62** |  | **58** | **58** |  | **58** | **58** |
| 0.03 |  |  |  |  |  |  |  | **39** |  |  | **110** |
| 0.075 |  |  |  |  |  |  |  | **126** |  |  | **158** |
| 0.15 |  |  |  |  |  |  |  | **172** |  | **80** | **130** |
| 0.3 | **91** | **220** |  | **69** | **512** |  | **0** | **235** |  | **108** | **223** |
| 0.75 | **94** | **289** |  | **93** | **930** |  | **71** | 84 |  | **187** | 312 |
| 1.5 | **169** | 384 |  | **122** | 1305 |  | **89** |  |  | 150 |  |
| 3 | **394** | 207 |  | **280** | 1962 |  | **277** |  |  |  |  |
| 6 | **555** | 325 |  | **364** | 1981 |  |  |  |  |  |  |
|  |  |  |  |  |  |  |  |  |  |  |  |
| Sample ID: | #11 | week 1 |  | #11 | week 2 |  | #12 | week 1 |  | #12 | week 2 |
| ml-eq./plate | U | H |  | U | H |  | U | H |  | U | H |
| 0 | **58** | **58** |  | **58** | **58** |  | **58** | **58** |  | **58** | **58** |
| 0.03 |  | **75** |  |  | **80** |  |  | **65** |  |  | **65** |
| 0.075 |  | **107** |  |  | **116** |  |  | **85** |  |  | **83** |
| 0.15 |  | **131** |  |  | **159** |  |  | **123** |  |  | **136** |
| 0.3 | **63** | **225** |  |  | **271** |  | **68** | 125 |  |  | 136 |
| 0.75 | **92** | 301 |  |  | 145 |  | **75** | 165 |  | **96** | 168 |
| 1.5 | **160** |  |  | **189** |  |  | **90** |  |  | **127** |  |
| 3 | **299** |  |  | **392** |  |  | **165** |  |  | **269** |  |
| 6 | **475** |  |  | 79 |  |  | **254** |  |  | **460** |  |
| 7.5 |  |  |  | 127 |  |  |  |  |  |  |  |
|  |  |  |  |  |  |  |  |  |  |  |  |
| Sample ID: | #13 | week 3 |  | #13 | week 4 |  |  |  |  |  |  |
| ml-eq./plate | U | H |  | U | H |  |  |  |  |  |  |
| 0 | **53** | **53** |  | **53** | **53** |  |  |  |  |  |  |
| 0.075 |  |  |  |  | **192** |  |  |  |  |  |  |
| 0.15 |  | **177** |  |  | **291** |  |  |  |  |  |  |
| 0.3 | **84** | **337** |  |  | **762** |  |  |  |  |  |  |
| 0.75 | **114** | 538 |  | **88** | **1495** |  |  |  |  |  |  |
| 1.5 | **226** | 581 |  | **150** | 1950 |  |  |  |  |  |  |
| 3 | **521** |  |  | **334** |  |  |  |  |  |  |  |
| 6 | **644** |  |  | **535** |  |  |  |  |  |  |  |

Table S4 – continued

Urine mutagenicity after consumption of the meal containing meat fried at high temperature^a^ together with dietary inhibitors^b^

| Sample ID: | #14 ^c^ | week 3 |  | #14 | week 4 |  |  |  |
| --- | --- | --- | --- | --- | --- | --- | --- | --- |
| ml-eq./plate | U | H |  | U | H |  |  |  |
| 0 | **53** ^d^ | **53** |  | **53** | **53** |  |  |  |
| 0.15 |  | **151** |  |  | **155** |  |  |  |
| 0.3 |  | **223** |  |  | **266** |  |  |  |
| 0.75 | **88** | 347 |  | **118** | 351 |  |  |  |
| 1.5 | **87** | 434 |  | **141** | 400 |  |  |  |
| 3 | **349** | 523 |  | **268** |  |  |  |  |
| 6 | **423** |  |  | **485** |  |  |  |  |
|  |  |  |  |  |  |  |  |  |
| Sample ID: | #15 | week 1 |  | #15 | week 2 |  |  |  |
| ml-eq./plate | U | H |  | U | H |  |  |  |
| 0 | **53^c^** | **53** |  | **53** | **53** |  |  |  |
| 0.075 |  | **119** |  |  | **57** |  |  |  |
| 0.15 |  | **241** |  |  | **133** |  |  |  |
| 0.3 | **33** | **681** |  | **82** | **199** |  |  |  |
| 0.75 | **44** | 1096 |  | **150** | 276 |  |  |  |
| 1.5 | **63** | 1005 |  |  | 411 |  |  |  |
| 3 | **141** |  |  |  |  |  |  |  |
| 6 | **181** |  |  | **494** |  |  |  |  |
|  |  |  |  |  |  |  |  |  |
| Sample ID: | #16 | week 1 |  | #16 | week 2 |  |  |  |
| ml-eq./plate | U | H |  | U | H |  |  |  |
| 0 | **53** | **53** |  | **53** | **53** |  |  |  |
| 0.15 |  | **106** |  |  | **76** |  |  |  |
| 0.3 |  | **228** |  | **77** | **141** |  |  |  |
| 0.75 | **93** | 159 |  | **98** | **218** |  |  |  |
| 1.5 |  | 279 |  |  | 238 |  |  |  |
| 6 | **432** |  |  | 291 |  |  |  |  |

^a^Beef and sausage patties were cooked at 250°C for 11 min on each side.

^b^Dietary inhibitors were cruciferous vegetables, yogurt, and chlorophyllin tablets.

^c^Indicates subject number. Subjects consumed diets containing meat cooked at high

temperature alone or with inhibitors for 2-week periods in a cross-over design. Counts for control (0

ml-eq./plate) plates are the average of 3 separate plates. All other concentrations were tested in single plates.

^d^Counts designated as within the linear portion of the dose-response curve are in bold type

U = unhydrolyzed extracts H = acid-hydroylzed extracts

Table S5

Urine creatinine values (µmoles/ml)^a^

|  |  |  |  |  |  |  |  |  |
| --- | --- | --- | --- | --- | --- | --- | --- | --- |

| Sample ID: |  |  |  |  |  |  |  |
| --- | --- | --- | --- | --- | --- | --- | --- |
| #1 week 1 | 10.29 | #5 week 1 | 10.19 | #9 week 1 | 12.59 | #13 week 1 | 15.26 |
| #1 week 2 | 9.64 | #5 week 2 | 12.33 | #9 week 2 | 13.27 | #13 week 2 | 20.19 |
| #1 week 3 | 10.85 | #5 week 3 | 14.44 | #9 week 3 | 14.30 | #13 week 3 | 4.99 |
| #1 week 4 | 9.67 | #5 week 4 | 12.55 | #9 week 4 | 17.29 | #13 week 4 | 12.73 |
| #2 week 1 | 12.74 | #6 week 1 | 19.81 | #10 week 1 | 10.62 | #14 week 1 | 10.57 |
| #2 week 2 | 17.35 | #6 week 2 | 20.57 | #10 week 2 | 13.66 | #14 week 2 | 7.83 |
| #2 week 3 | 14.58 | #6 week 3 | 13.66 | #10 week 3 | 11.71 | #14 week 3 | 9.13 |
| #2 week 4 | 12.93 | #6 week 4 | 15.37 | #10 week 4 | 18.98 | #14 week 4 | 11.54 |
| #3 week 1 | 11.07 | #7 week 1 | 24.25 | #11 week 1 | 11.20 | #15 week 1 | 16.12 |
| #3 week 2 | 15.75 | #7 week 2 | 26.09 | #11 week 2 | 15.13 | #15 week 2 | 10.68 |
| #3 week 3 | 11.84 | #7 week 3 | 21.67 | #11 week 3 | 17.23 | #15 week 3 | 8.25 |
| #3 week 4 | 13.59 | #7 week 4 | 14.73 | #11 week 4 | 16.77 | #15 week 4 | 12.22 |
| #4 week 1 | 9.13 | #8 week 1 | 6.06 | #12 week 1 | 7.77 | #16 week 1 | 14.57 |
| #4 week 2 | 11.47 | #8 week 2 | 6.74 | #12 week 2 | 8.41 | #16 week 2 | 13.68 |
| #4 week 3 | 6.36 | #8 week 3 | 10.51 | #12 week 3 | 5.99 | #16 week 3 | 13.24 |
| #4 week 4 | 8.01 | #8 week 4 | 9.18 | #12 week 4 | 8.11 | #16 week 4 | 6.63 |

|  | | |  | |  |  |  |  |  |  |
| --- | --- | --- | --- | --- | --- | --- | --- | --- | --- | --- |
| ^a^ To estimate mutagenic potency for urine as rev/µmoles creatinine, we fit models using doses expressed in µmoles creatinine. These doses were calculated by multiplying the dose value in ml-eq/plate from Tables S2-S4 by the subject- and week-specific creatinine values in this table.  Table S6  Fecal mutagenicity after consumption of the meal containing meat fried at low temperature^a^ | | | | | | | | | | |
|  |  | |  | |  |  |  |  |  |  |
| Sample ID: | **#1^b^** | |  | |  |  |  |  |  |  |
|  | Expt 1 | | |  | | Expt 2 |  |  | Expt 3 |  |
| µg EOM/plate | **U** | **H** | |  | | **U** | **H** |  | **U** | **H** |
| 0 | **62^c^** | **62** | |  | | **65** | **65** |  | **62** | **62** |
| 40 | **54** | **60** | |  | | **70** | **68** |  | **61** | **59** |
| 100 | **84** | **88** | |  | | **81** | **94** |  | **82** | **76** |
| 400 | **118** | 0 | |  | | **89** | **92** |  | 54 | **86** |
|  |  |  | |  | |  |  |  |  |  |
| Sample ID: | **#2** |  | |  | |  |  |  |  |  |
|  | Expt 1 |  | |  | | Expt 2 |  |  | Expt 3 |  |
|  | **U** | **H** | |  | | **U** | **H** |  | **U** | **H** |
| 0 | **60** | **60** | |  | | **59** | **59** |  | **55** | **55** |
| 40 | **45** | **61** | |  | | **60** | **54** |  | **73** | **67** |
| 100 | **65** | **104** | |  | | **78** | **85** |  | **86** | **82** |
| 400 | **93** | **88** | |  | | **90** | **83** |  | **125** | **104** |
|  |  |  | |  | |  |  |  |  |  |
| Sample ID: | **#3** |  | |  | |  |  |  |  |  |
|  | Expt 1 |  | |  | | Expt 2 |  |  | Expt 3 |  |
| µg EOM/plate | **U** | **H** | |  | | **U** | **H** |  | **U** | **H** |
| 0 | **60** | **60** | |  | | **59** | **59** |  | **55** | **55** |
| 40 | **65** | **59** | |  | | **56** | **68** |  | **68** | **55** |
| 100 | **98** | **92** | |  | | **99** | **101** |  | **101** | **119** |
| 400 | **125** | **91** | |  | | **136** | **101** |  | **135** | **119** |
|  |  |  | |  | |  |  |  |  |  |
| Sample ID: | **#4** |  | |  | |  |  |  |  |  |
|  | Expt 1 | | |  | | Expt 2 |  |  | Expt 3 |  |
| µg EOM/plate | **U** | **H** | |  | | **U** | **H** |  | **U** | **H** |
| 0 | **54** | **54** | |  | | **65** | **65** |  | **62** | **62** |
| 40 | **52^d^** | **72^d^** | |  | | **62^d^** | **54^d^** |  | **62** | **53** |
| 100 | **79^d^** | **103^d^** | |  | | **82^d^** | **116^d^** |  | **81** | **88** |
| 400 | **134^d^** | **150^d^** | |  | | **112^d^** | **120^d^** |  | **66** | **94** |
|  |  |  | |  | |  |  |  |  |  |
| Sample ID: | **#5** |  | |  | |  |  |  |  |  |
|  | Expt 1 |  | |  | | Expt 2 |  |  | Expt 3 |  |
| µg EOM/plate | **U** | **H** | |  | | **U** | **H** |  | **U** | **H** |
| 0 | **60** | **60** | |  | | **59** | **59** |  | **55** | **55** |
| 40 | **56** | **59** | |  | | **65** | **71** |  | **67** | **76** |
| 100 | **63** | **81** | |  | | **72** | **95** |  | **76** | **84** |
| 400 | 53 | **105** | |  | | 60 | 102 |  | **111** | 98 |
|  |  |  | |  | |  |  |  |  |  |
|  |  |  | |  | |  |  |  |  |  |
|  |  |  | |  | |  |  |  |  |  |
| Table S6 – continued  Fecal mutagenicity after consumption of the meal containing meat fried at low temperature^a^ | | | | | | | | | | |
|  |  |  | |  | |  |  |  |  |  |
| Sample ID: | **#6** |  | |  | |  |  |  |  |  |
|  | Expt 1 | | |  | | Expt 2 |  |  | Expt 3 |  |
| µg EOM/plate | **U** | **H** | |  | | **U** | **H** |  | **U** | **H** |
| 0 | **62** | **62** | |  | | **65** | **65** |  | **62** | **62** |
| 40 | **55** | **60** | |  | | **69^d^** | **67^d^** |  | **69** | **58** |
| 100 | **69** | **86** | |  | | **74^d^** | **67^d^** |  | **87** | **83** |
| 400 | 0 | 0 | |  | | **80^d^** | **112^d^** |  | **102** | **137** |
|  |  |  | |  | |  |  |  |  |  |
|  |  |  | |  | |  |  |  |  |  |
| Sample ID: | **#7** |  | |  | |  |  |  |  |  |
|  | Expt 1 |  | |  | | Expt 2 |  |  | Expt 3 |  |
| µg EOM/plate | **U** | **H** | |  | | **U** | **H** |  | **U** | **H** |
| 0 | **60** | **60** | |  | | **59** | **59** |  | **55** | **55** |
| 40 | **59** | **42** | |  | | **56** | **79** |  | **79** | **65** |
| 100 | **72** | **76** | |  | | **58** | **82** |  | **91** | **82** |
| 400 | **91** | **76** | |  | | **60** | **64** |  | **95** | **102** |
|  |  |  | |  | |  |  |  |  |  |
|  |  |  | |  | |  |  |  |  |  |
| Sample ID: | **#8** |  | |  | |  |  |  |  |  |
|  | Expt 1 |  | |  | | Expt 2 |  |  | Expt 3 |  |
| µg EOM/plate | **U** | **H** | |  | | **U** | **H** |  | **U** | **H** |
| 0 | **60** | **60** | |  | | **59** | **59** |  | **55** | **55** |
| 40 | **46** | **60** | |  | | **55** | **49** |  | **68** | **69** |
| 100 | **49** | **61** | |  | | **58** | **61** |  | **94** | **86** |
| 400 | 55 | **75** | |  | | **72** | **70** |  | **138** | **97** |

^a^Beef and sausage patties were cooked at 100°C for 10 min on each side.

^b^Indicates subject number. Subjects consumed diets containing meat cooked at either low or high temperature for 2-week periods in a cross-over design.

^c^Counts designated as within the linear portion of the dose-response curve are in bold type. These are the mean values for 2 plates/experiment except where noted. Each extract was tested in 3 independent experiments. Assays with the same zero dose mean count were conducted on the same day.

U = unhydrolyzed extracts H = acid-hydroylzed extracts

^d^Extracts tested in single plates.

| Table S7 | | | | | | | | |
| --- | --- | --- | --- | --- | --- | --- | --- | --- |
|  | |  |  |  |  |  |  |  |
| Fecal mutagenicity after consumption of the meal containing meat fried at high temperature^a^ | | | | | | | | |
|  |  |  |  |  |  |  |  |  |
| Sample ID: | **#1^b^** |  |  |  |  |  |  |  |
|  | Expt 1 |  |  | Expt 2 |  |  | Expt 3 |  |
| µg EOM/plate | **U** | **H** |  | **U** | **H** |  | **U** | **H** |
| 0 | **60^c^** | **60** |  | **59** | **59** |  | **55** | **55** |
| 40 | **61** | **64** |  | **61** | **63** |  | **63** | **71** |
| 100 | **82** | **94** |  | **86** | **110** |  | **102** | **107** |
| 400 | **97** | **146** |  | 96 | **143** |  | **115** | **140** |
|  |  |  |  |  |  |  |  |  |
| Sample ID: | **#2** |  |  |  |  |  |  |  |
|  | Expt 1 | |  | Expt 2 |  |  | Expt 3 |  |
| µg EOM/plate | **U** | **H** |  | **U** | **H** |  | **U** | **H** |
| 0 | **54** | **54** |  | **65** | **65** |  | **62** | **62** |
| 40 | **60^d^** | **75^d^** |  | **97^d^** | **66^d^** |  | **72** | **70** |
| 100 | **70^d^** | **131^d^** |  | **107^d^** | **173^d^** |  | **124** | **119** |
| 400 | **113^d^** | **216^d^** |  | **209^d^** | **192^d^** |  | **165** | **170** |
|  |  |  |  |  |  |  |  |  |
| Sample ID: | **#3** |  |  |  |  |  |  |  |
|  | Expt 1 |  |  | Expt 2 |  |  | Expt 3 |  |
| µg EOM/plate | **U** | **H** |  | **U** | **H** |  | **U** | **H** |
| 0 | **60** | **60** |  | **59** | **59** |  | **55** | **55** |
| 40 | **50** | **60** |  | **59** | **75** |  | **55** | **74** |
| 100 | **66** | **136** |  | **73** | **160** |  | **76** | **107** |
| 400 | **82** | 167 |  | **84** | 212 |  | **93** | 161 |
|  |  |  |  |  |  |  |  |  |
| Sample ID: | **#4** |  |  |  |  |  |  |  |
|  | Expt 1 |  |  | Expt 2 |  |  | Expt 3 |  |
| µg EOM/plate | **U** | **H** |  | **U** | **H** |  | **U** | **H** |
| 0 | **60** | **60** |  | **59** | **59** |  | **55** | **55** |
| 40 | **74** | **71** |  | **62** | **79** |  | **75** | **81** |
| 100 | **102** | **137** |  | **83** | **154** |  | **116** | **148** |
| 400 | 116 | 171 |  | **77** | 193 |  | 118 | 215 |
|  |  |  |  |  |  |  |  |  |
| Sample ID: | **#5** |  |  |  |  |  |  |  |
|  | Expt 1 |  |  | Expt 2 |  |  | Expt 3 |  |
| µg EOM/plate | **U** | **H** |  | **U** | **H** |  | **U** | **H** |
| 0 | **60** | **60** |  | **59** | **59** |  | **55** | **55** |
| 40 | **55** | **62** |  | **52** | **77** |  | **66** | **78** |
| 100 | **75** | **163** |  | **61** | **153** |  | **85** | **136** |
| 400 | **80** | 218 |  | **88** | 224 |  | **122** | 210 |
|  |  |  |  |  |  |  |  |  |
|  |  |  |  |  |  |  |  |  |
| Table S7 - continued | | | | | | | | |
| Sample ID: | **#6** |  |  |  |  |  |  |  |
|  | Expt 1 |  |  | Expt 2 |  |  | Expt 3 |  |
| µg EOM/plate | **U** | **H** |  | **U** | **H** |  | **U** | **H** |
| 0 | **60** | **60** |  | **59** | **59** |  | **55** | **55** |
| 40 | **52** | **69** |  | **68** | **66** |  | **72** | **73** |
| 100 | **59** | **109** |  | **73** | **130** |  | **58** | **119** |
| 400 | **63** | **185** |  | **87** | **210** |  | **127** | **166** |
|  |  |  |  |  |  |  |  |  |
| Sample ID: | **#7** |  |  |  |  |  |  |  |
|  | Expt 1 | |  |  |  |  |  |  |
| µg EOM/plate | **U** | **H** |  | **U** | **H** |  | **U** | **H** |
| 0 | **62** | **62** |  | **65** | **65** |  | **62** | **62** |
| 40 | **73** | **75** |  | **66^d^** | **100^d^** |  | **63** | **74** |
| 100 | **70** | **92** |  | **81^d^** | **130^d^** |  | **60** | **158** |
| 400 | **70** | 82 |  | **101^d^** | 220^d^ |  | **94** | 197 |
|  |  |  |  |  |  |  |  |  |
| Sample ID: | **#8** |  |  |  |  |  |  |  |
|  | Expt 1 |  |  | Expt 2 |  |  | Expt 3 |  |
| µg EOM/plate | **U** | **H** |  | **U** | **H** |  | **U** | **H** |
| 0 | **60** | **60** |  | **59** | **59** |  | **55** | **55** |
| 40 | **53** | **45** |  | **65** | **71** |  | **66** | **79** |
| 100 | **52** | **115** |  | **74** | **139** |  | **95** | **141** |
| 400 | **58** | 151 |  | **91** | 176 |  | **97** | 179 |
|  |  |  |  |  |  |  |  |  |
| Sample ID: | **#9** |  |  |  |  |  |  |  |
|  | Expt 1 |  |  | Expt 2 |  |  | Expt 3 |  |
| µg EOM/plate | **U** | **H** |  | **U** | **H** |  | **U** | **H** |
| 0 | **60** | **60** |  | **59** | **59** |  | **55** | no data |
| 40 | **63** | **77** |  | **79** | **77** |  | **65** |  |
| 100 | **88** | **119** |  | **112** | **197** |  | **101** |  |
| 400 | **109** | 182 |  | 122 | 234 |  |  |  |
|  |  |  |  |  |  |  |  |  |
| Sample ID: | **#10** |  |  |  |  |  |  |  |
|  | Expt 1 |  |  | Expt 2 |  |  | Expt 3 |  |
| µg EOM/plate | **U** | **H** |  | **U** | **H** |  | **U** | **H** |
| 0 | **60** | **60** |  | **59** | **59** |  | **55** | **55** |
| 40 | **62** | **58** |  | **71** | **87** |  | **88** | **70** |
| 100 | **67** | **107** |  | **114** | **136** |  | **97** | **128** |
| 400 | **129** | 143 |  | 120 | 173 |  | 92 | 157 |
|  |  |  |  |  |  |  |  |  |
| Sample ID: | **#11** |  |  |  |  |  |  |  |
|  | Expt 1 |  |  | Expt 2 |  |  | Expt 3 |  |
| µg EOM/plate | **U** | **H** |  | **U** | **H** |  | **U** | **H** |
| 0 | **60** | **60** |  | **59** | **59** |  | **55** | **55** |
| 40 | **44** | **48** |  | **63** | **73** |  | **72** | **76** |
| 100 | **68** | **103** |  | **99** | **149** |  | **88** | **124** |
| 400 | **75** | **147** |  | **95** | 189 |  | **126** | 198 |
| Table S7 - continued | | | | | | | | |
|  |  |  |  |  |  |  |  |  |
| Sample ID: | **#12** |  |  |  |  |  |  |  |
|  | Expt 1 |  |  | Expt 2 |  |  | Expt 3 |  |
| µg EOM/plate | **U** | **H** |  | **U** | **H** |  | **U** | **H** |
| 0 | **60** | **60** |  | **59** | **59** |  | **55** | **55** |
| 40 | **40** | **56** |  | **66** | **69** |  | **76** | **94** |
| 100 | **61** | **102** |  | **77** | **81** |  | **76** | **118** |
| 400 | **58** | **140** |  | **92** | **191** |  | **66** | **152** |
|  |  |  |  |  |  |  |  |  |
| Sample ID: | **#13** |  |  |  |  |  |  |  |
|  | Expt 1 |  |  | Expt 2 |  |  | Expt 3 |  |
| µg EOM/plate | **U** | **H** |  | **U** | **H** |  | **U** | **H** |
| 0 | **60** | **60** |  | **59** | **59** |  | **55** | **55** |
| 40 | **51** | **62** |  | **55** | **53** |  | **69** | **141** |
| 100 | **99** | **143** |  | **87** | **115** |  | **112** | **87** |
| 400 | **122** | 202 |  | **82** | **190** |  | 124 | 192 |
|  |  |  |  |  |  |  |  |  |
| Sample ID: | **#14** |  |  |  |  |  |  |  |
|  | Expt 1 |  |  | Expt 2 |  |  | Expt 3 |  |
| µg EOM/plate | **U** | **H** |  | **U** | **H** |  | **U** | **H** |
| 0 | **60** | **60** |  | **59** | **59** |  | **55** | **55** |
| 40 | **53** | **52** |  | **70** | **85** |  | **80** | **73** |
| 100 | **63** | **139** |  | **81** | **176** |  | **95** | **109** |
| 400 | **71** | **230** |  | **126** | 214 |  | **142** | **202** |
|  |  |  |  |  |  |  |  |  |
| Sample ID: | **#15** |  |  |  |  |  |  |  |
|  | Expt 1 |  |  | Expt 2 |  |  | Expt 3 |  |
| µg EOM/plate | **U** | **H** |  | **U** | **H** |  | **U** | **H** |
| 0 | **60** | **60** |  | **59** | **59** |  | **55** | **55** |
| 40 | **39** | **66** |  | **70** | **50** |  | **70** | **74** |
| 100 | **63** | **140** |  | **165** | **80** |  | **105** | **151** |
| 400 | **60** | 172 |  | 224 | **84** |  | 131 | 167 |
|  |  |  |  |  |  |  |  |  |
| Sample ID: | **#16** |  |  |  |  |  |  |  |
|  | Expt 1 |  |  | Expt 2 |  |  | Expt 3 |  |
| µg EOM/plate | **U** | **H** |  | **U** | **H** |  | **U** | **H** |
| 0 | **60** | no data |  | **59** | **59** |  | **55** | **55** |
| 40 | **50** |  |  | **63** | **72** |  | **74** | **65** |
| 100 | **50** |  |  | **78** | **148** |  | **122** | **145** |
| 400 | **66** |  |  | **100** | 201 |  | 152 | 172 |

^a^Beef and sausage patties were cooked at 250°C for 11 min on each side.

^b^Indicates subject number. Subjects consumed diets containing meat cooked at either low or high temperature for 2-week periods in a cross-over design.

^c^Counts designated as within the linear portion of the dose-response curve are in bold type. These are mean values for 2 plates/experiment except where noted. Each extract was tested in 3 independent experiments.

^d^Extracts tested in single plates.

U = unhydrolyzed extracts H = acid-hydrolyzed extracts

| Table S8 | | | | | | | | |
| --- | --- | --- | --- | --- | --- | --- | --- | --- |
|  | |  |  |  |  |  |  |  |
| Fecal mutagenicity after consumption of the meal containing meat fried at high temperature^a^ together with dietary inhibitors^b^ | | | | | | | | |
|  |  |  |  |  |  |  |  |  |
| Sample ID: | **#9^c^** |  |  |  |  |  |  |  |
|  | Expt 1 |  |  | Expt 2 |  |  | Expt 3 |  |
| µg EOM/plate | **U** | **H** |  | **U** | **H** |  | **U** | **H** |
| 0 | **60^d^** | **60** |  | **59** | **59** |  | **55** | **55** |
| 40 | **64** | **46** |  | **65** | **63** |  | **66** | **77** |
| 100 | **71** | **106** |  | **88** | **151** |  | **110** | **173** |
| 400 | **86** | **207** |  | **92** | **287** |  | **112** | **235** |
|  |  |  |  |  |  |  |  |  |
| Sample ID: | **#10** |  |  |  |  |  |  |  |
|  | Expt 1 |  |  | Expt 2 |  |  | Expt 3 |  |
| µg EOM/plate | **U** | **H** |  | **U** | **H** |  | **U** | **H** |
| 0 | **60** | **60** |  | **59** | **59** |  | **55** | **55** |
| 40 | **54** | **67** |  | **65** | **73** |  | **61** | **79** |
| 100 | **77** | **108** |  | **98** | **119** |  | **96** | **98** |
| 400 | **97** | **144** |  | **119** | **188** |  | 93 | **127** |
|  |  |  |  |  |  |  |  |  |
| Sample ID: | **#11** |  |  |  |  |  |  |  |
|  | Expt 1 |  |  | Expt 2 |  |  | Expt 3 |  |
| µg EOM/plate | **U** | **H** |  | **U** | **H** |  | **U** | **H** |
| 0 | **60** | **60** |  | **59** | **59** |  | **55** | **55** |
| 40 | **48** | **61** |  | **44** | **52** |  | **76** | **72** |
| 100 | **76** | **53** |  | **72** | **122** |  | **83** | **130** |
| 400 | **80** | 132 |  | **70** | 157 |  | **96** | 165 |
|  |  |  |  |  |  |  |  |  |
| Sample ID: | **#12** |  |  |  |  |  |  |  |
|  | Expt 1 |  |  | Expt 2 |  |  | Expt 3 |  |
| µg EOM/plate | **U** | **H** |  | **U** | **H** |  | **U** | **H** |
| 0 | **60** | **60** |  | **59** | **59** |  | **55** | **55** |
| 40 | **58** | **67** |  | **64** | **68** |  | **66** | **82** |
| 100 | **75** | **92** |  | **72** | **117** |  | **72** | **140** |
| 400 | **73** | **214** |  | **73** | **176** |  | **84** | **232** |
|  |  |  |  |  |  |  |  |  |
| Sample ID: | **#13** |  |  |  |  |  |  |  |
|  | Expt 1 |  |  | Expt 2 |  |  | Expt 3 |  |
| µg EOM/plate | **U** | **H** |  | **U** | **H** |  | **U** | **H** |
| 0 | **60** | **60** |  | **59** | **59** |  | **55** | **55** |
| 40 | **59** | **60** |  | **65** | **68** |  | **63** | **79** |
| 100 | **55** | **121** |  | **77** | **123** |  | **69** | **102** |
| 400 | **61** | **137** |  | **82** | **182** |  | **102** | **136** |
|  |  |  |  |  |  |  |  |  |
|  |  |  |  |  |  |  |  |  |
| Table S8 – continued  Fecal mutagenicity after consumption of the meal containing meat fried at high temperature^a^ together with dietary inhibitors^b^ | | | | | | | | |
| Sample ID: | **#14** |  |  |  |  |  |  |  |
|  | Expt 1 |  |  | Expt 2 |  |  | Expt 3 |  |
| µg EOM/plate | **U** | **H** |  | **U** | **H** |  | **U** | **H** |
| 0 | **60** | **60** |  | **59** | **59** |  | **55** | **55** |
| 40 | **41** | **56** |  | **61** | **66** |  | **60** | **65** |
| 100 | **70** | **119** |  | **75** | **137** |  | **112** | **158** |
| 400 | **75** | **185** |  | 73 | **193** |  | **139** | 169 |
|  |  |  |  |  |  |  |  |  |
| Sample ID: | **#15** |  |  |  |  |  |  |  |
|  | Expt 1 |  |  | Expt 2 |  |  | Expt 3 |  |
| µg EOM/plate | **U** | **H** |  | **U** | **H** |  | **U** | **H** |
| 0 | **60** | **60** |  | **59** | **59** |  | **55** | **55** |
| 40 | **46** | **86** |  | **56** | **80** |  | **75** | **71** |
| 100 | **75** | **157** |  | **73** | **157** |  | **108** | **124** |
| 400 | **75** | **211** |  | 74 | 203 |  | **125** | **191** |
|  |  |  |  |  |  |  |  |  |
| Sample ID: | **#16** |  |  |  |  |  |  |  |
|  | Expt 1 |  |  | Expt 2 |  |  | Expt 3 |  |
| µg EOM/plate | **U** | **H** |  | **U** | **H** |  | **U** | **H** |
| 0 | **60** | **60** |  | **59** | **59** |  | **55** | **55** |
| 40 | **60** | **63** |  | **67** | **69** |  | **75** | **76** |
| 100 | **73** | **107** |  | **106** | **170** |  | **148** | **155** |
| 400 | **81** | **183** |  | 102 | 241 |  | 127 | 162 |

^a^Beef and sausage patties were cooked at 250°C for 11 min on each side.

^b^Dietary inhibitors were cruciferous vegetables, yogurt, and chlorophyllin tablets.

^c^Indicates subject number. Subjects consumed diets containing meat cooked at high temperature alone or with inhibitors for 2-week periods in a cross-over design.

^d^Counts designated as within the linear portion of the dose-response curve are in bold type. These are mean values for 2 plates/experiment. Each extract was tested in 3 independent experiments.

U = unhydrolyzed extracts H = acid-hydroylzed extracts

Table S9

% Extractable organic matter (%EOM)^a^

| Sample ID: | Unhydrolyzed | Hydrolyzed | Sample ID: | Unhydrolyzed | Hydrolyzed |
| --- | --- | --- | --- | --- | --- |
| #1 week 2 | 9.70 | 10.48 | #9 week 2 | 6.80 | 6.42 |
| #1 week 4 | 5.65 | 5.98 | #9 week 4 | 6.11 | 5.48 |
| #2 week 2 | 8.52 | 8.53 | #10 week 2 | 11.31 | 12.46 |
| #2 week 4 | 7.43 | 8.80 | #10 week 4 | 9.30 | 9.87 |
| #3 week 2 | 9.13 | 7.50 | #11 week 2 | 11.53 | 12.37 |
| #3 week 4 | 7.58 | 7.28 | #11 week 4 | 10.49 | 10.19 |
| #4 week 2 | 5.55 | 5.91 | #12 week 2 | 8.50 | 8.52 |
| #4 week 4 | 5.58 | 7.04 | #12 week 4 | 10.01 | 12.74 |
| #5 week 2 | 7.06 | 8.41 | #13 week 2 | 8.84 | 8.32 |
| #5 week 4 | 7.40 | 7.83 | #13 week 4 | 9.00 | 8.84 |
| #6 week 2 | 5.89 | 8.70 | #14 week 2 | 10.36 | 9.73 |
| #6 week 4 | 11.25 | 9.12 | #14 week 4 | 8.31 | 9.70 |
| #7 week 2 | 6.68 | 5.72 | #15 week 2 | 9.84 | 9.32 |
| #7 week 4 | 7.29 | 6.89 | #15 week 4 | 10.13 | 10.66 |
| #8 week 2 | 9.03 | 7.84 | #16 week 2 | 8.75 | 9.55 |
| #8 week 4 | 7.83 | 9.28 | #16 week 4 | 8.69 | 9.31 |

^a^To estimate mutagenic potency for feces as rev/mg lyophilized stool, we fit models using doses expressed in mg lyophilized stool. These doses were calculated by dividing the dose value in µg EOM/plate from Tables S6-S8 by subject- and week-specific %EOM values from this table expressed as a proportion (%EOM/100) and then dividing the result by 1000 to change µg to mg.

Table S10

Comet assay results for white blood cells expressed as tail moment (TM)

| **Sample ID:** | **Diet** | **Median TM**  **Week**  **1 2 3 4** | | | | **Mean** |  |
| --- | --- | --- | --- | --- | --- | --- | --- |
| #1^a^ | LT | 0.51^b^ | 0.33 |  |  | 0.42 |  |
|  | HT |  |  | 0.29 | 0.16 | 0.21 |  |
|  |  |  |  |  |  |  |  |
| #2 | LT |  |  | 0.30 | 0.11 | 0.21 |  |
|  | HT | 0.21 | 0.63 |  |  | 0.41 |  |
|  |  |  |  |  |  |  |  |
| #3 | LT | 0.23 | 0.48 |  |  | 0.35 |  |
|  | HT |  |  | 0.29 | 0.08 | 0.18 |  |
|  |  |  |  |  |  |  |  |
| #4 | LT |  |  | 0.23 | 0.16 | 0.20 |  |
|  | HT | 0.30 | 0.61 |  |  | 0.45 |  |
|  |  |  |  |  |  |  |  |
| #5 | LT | 0.85 | 1.06 |  |  | 0.95 |  |
|  | HT |  |  | 0.53 | 0.09 | 0.31 |  |
|  |  |  |  |  |  |  |  |
| #6 | LT | 1.36 | 1.82 |  |  | 1.59 |  |
|  | HT |  |  | 0.40 | 0.09 | 0.24 |  |
|  |  |  |  |  |  |  |  |
| #7 | LT |  |  | 0.35 | 0.09 | 0.22 |  |
|  | HT | 1.21 | 1.21 |  |  | 1.21 |  |
|  |  |  |  |  |  |  |  |
| #8 | LT |  |  | 0.84 | 0.16 | 0.50 |  |
|  | HT | 0.92 | 1.73 |  |  | 1.32 |  |
|  |  |  |  |  |  |  |  |
| #9 | HT | 0.06 | 0.40 |  |  | 0.23 |  |
|  | HT+I |  |  | 0.20 | 0.12 | 0.16 |  |
|  |  |  |  |  |  |  |  |
| #10 | HT | 0.07 | 0.38 |  |  | 0.23 |  |
|  | HT+I |  |  | 0.15 | 0.10 | 0.12 |  |
|  |  |  |  |  |  |  |  |
| #11 | HT |  |  | 0.15 | 0.12 | 0.14 |  |
|  | HT+I | 0.07 | 0.33 |  |  | 0.2 |  |
|  |  |  |  |  |  |  |  |
| #12 | HT |  |  | 0.18 | 0.19 | 0.19 |  |
|  | HT+I | 0.07 | 0.30 |  |  | 0.19 |  |
|  |  |  |  |  |  |  |  |
| #13 | HT | 0.14 | 0.13 |  |  | 0.13 |  |
|  | HT+I |  |  | 0.14 | 0.07 | 0.1 |  |
|  |  |  |  |  |  |  |  |
| #14 | HT | 0.11 | 0.16 |  |  | 0.13 |  |
|  | HT+I |  |  | 0.13 | 0.08 | 0.10 |  |
|  |  |  |  |  |  |  |  |
| #15 | HT | 0.14 | 0.05 |  |  | 0.09 |  |
|  | HT+I |  |  | 0.14 | 0.20 | 0.17 |  |
|  |  |  |  |  |  |  |  |
| #16 | HT |  |  | 0.12 | 0.05 | 0.08 |  |
|  | HT+I | 0.14 | 0.12 |  |  | 0.13 |  |

^a^indicates subject number. Subjects consumed diets containing meat cooked at either low (LT) or high temperature (HT) or the HT diet with inhibitors (HT+I) for 2-week periods in a cross-over design.

^b^Average of 4 slide-specific medians, each based on 50 cells/slide (a total of 200 cells/week/subject).

Table S11

Comet assay results for rectal cells expressed as tail moment (TM)

| **Sample ID:** | **Diet** | **Median TM**  **Week**  **1 2 3 4** | | | | **Mean** |  |
| --- | --- | --- | --- | --- | --- | --- | --- |
| #1^a^ | LT | 5.10^b^ | 13.99 |  |  | 9.54 |  |
|  | HT |  |  | 14.32 | 11.45 | 12.88 |  |
|  |  |  |  |  |  |  |  |
| #2 | LT |  |  | 10.10 | 7.89 | 8.99 |  |
|  | HT | 7.79 | 6.64 |  |  | 9.76 |  |
|  |  |  |  |  |  |  |  |
| #3 | LT | 9.09 | 6.95 |  |  | 8.02 |  |
|  | HT |  |  | 10.87 | 5.97 | 9.38 |  |
|  |  |  |  |  |  |  |  |
| #4 | LT |  |  | 9.83 | 7.56 | 8.69 |  |
|  | HT | 20.17 | 5.88 |  |  | 13.02 |  |
|  |  |  |  |  |  |  |  |
| #5 | LT | 3.68 | 5.08 |  |  | 4.38 |  |
|  | HT |  |  | 13.35 | 7.78 | 10.56 |  |
|  |  |  |  |  |  |  |  |
| #6 | LT | 2.46 | 6.74 |  |  | 4.60 |  |
|  | HT |  |  | 2.70 | 2.42 | 2.56 |  |
|  |  |  |  |  |  |  |  |
| #7 | LT |  |  | 9.88 | 7.28 | 8.58 |  |
|  | HT | 3.72 | 11.00 |  |  | 7.36 |  |
|  |  |  |  |  |  |  |  |
| #8 | LT |  |  | 3.47 | 3.01 | 3.24 |  |
|  | HT | 5.58 | 8.70 |  |  | 7.14 |  |
|  |  |  |  |  |  |  |  |
| #9 | HT | 4.40 | 3.74 |  |  | 4.07 |  |
|  | HT+I |  |  | 1.87 | 2.10 | 1.99 |  |
|  |  |  |  |  |  |  |  |
| #10 | HT | 5.50 | 4.25 |  |  | 4.87 |  |
|  | HT+I |  |  | 3.03 | 2.24 | 2.63 |  |
|  |  |  |  |  |  |  |  |
| #11 | HT |  |  | 6.88 | 4.53 | 5.71 |  |
|  | HT+I | 12.08 | 4.74 |  |  | 8.41 |  |
|  |  |  |  |  |  |  |  |
| #12 | HT |  |  | 3.52 | 5.77 | 4.64 |  |
|  | HT+I | 3.08 | 2.21 |  |  | 2.64 |  |
|  |  |  |  |  |  |  |  |
| #13 | HT | 3.77 | 12.06 |  |  | 7.91 |  |
|  | HT+I |  |  | 2.37 | 2.10 | 2.23 |  |
|  |  |  |  |  |  |  |  |
| #14 | HT | 5.92 | 9.34 |  |  | 7.63 |  |
|  | HT+I |  |  | 1.64 | 2.55 | 2.10 |  |
|  |  |  |  |  |  |  |  |
| #15 | HT |  |  | 6.72 | 3.44 | 5.08 |  |
|  | HT+I | 1.05 | 1.51 |  |  | 1.28 |  |
|  |  |  |  |  |  |  |  |
| #16 | HT |  |  | 8.07 | 1.36 | 4.72 |  |
|  | HT+I | 1.30 | 5.51 |  |  | 3.41 |  |

^a^indicates subject number. Subjects consumed diets containing meat cooked at either low (LT) or high temperature (HT) or the HT diet with inhibitors (HT+I) for 2-week periods in a cross-over design.

^b^Average of 4 slide-specific medians, each based on 50 cells/slide (a total of 200 cells/week/subject).
