# Supplementary material for: Inhibition of Fried Meat-Induced Colorectal DNA Damage and Altered Systemic Genotoxicity in Humans by Crucifera, Chlorophyllin, and Yogurt
Source: PLoS One. 2011 Apr 25;6(4):e18707. doi: 10.1371/journal.pone.0018707 (PMC3081825; doi:10.1371/journal.pone.0018707)
Supplement: Protocol S1 — Trial Protocol. (DOC) [file pone.0018707.s003.doc]

Master Protocol

Inhibition of Fried Meat-induced DNA Damage: A Dietary Intervention Study

GCRC#2159

Principal Investigator: Jack Taylor, MD, PhD

Co-Principal Investigators: Daniel Shaughnessy, PhD, Lisa Gangarosa, MD, Robert Sandler, MD, MPH

# Purpose and Rationale

Colorectal cancer is one of the most common forms of cancer worldwide and is the second leading cause of cancer death in the United States. Given the frequency of occurrence and the poor prognosis for many afflicted individuals, prevention of this disease has been a major public health priority for the past few decade. However, evidence from epidemiological studies has yet to explain the tremendous excess risk in developed countries, a trend that has been tentatively attributed to lifestyle and dietary factors. In the past few years, the consumption of red meat in particular, and other meat products, has attracted considerable attention in epidemiological, clinical, and experimental studies, with an increasing focus on a certain class of compounds found in cooked meat, heterocyclic amines. Heterocyclic amines are substances formed through pyrolysis of amino acids and creatine when meats are cooked at high temperature, particularly by pan-frying. Heterocyclic amine levels increase with cooking temperature, with the type and shape of the cooked piece of meat, with the degree of browning on the surface, and with the cooking method. In several epidemiological studies, including studies from Harvard, the International Agency for Research on Cancer (IARC), and the National Cancer Institute (NCI), individuals with long-term exposure to heterocyclic amines in their diet had an increased risk of colorectal cancers and colorectal adenomas, respectively. However, not all studies of heterocyclic amines in humans have shown a positive association with colorectal cancer risk, and clear consensus regarding this association is lacking.

Based on the implications from these epidemiological studies, researchers at NCI have recently conducted a series of controlled feeding studies aimed at relating short-term exposure to heterocyclic amines through fried meat consumption to transient changes in urine mutagenicity levels (Peters et al., 2003). These studies provide evidence that meat fried at high temperatures results in significant short-term increases in exposure to mutagens. However, whereas urine mutagenicity appears to be a good short-term measure of exposure to heterocyclic amines in the diet, it is unclear as to whether these compounds induce genetic damage in colonic epithelial cells in humans. Against this background, we propose conducting a feeding study that includes the examination of colon tissue for evidence of transient DNA damage detected in the single cell gel electrophoresis (comet) assay following experimental intake of fried meat. This study will provide more direct evidence than previous studies as to whether genetic damage in colon epithelium and lymphocytes is related to dietary intake of heterocyclic amines. The use of the comet assay to detect the effects of diet on transient DNA damage is novel in this type of controlled feeding study, particularly with respect to monitoring effects of diet on colon epithelial cells. We will also monitor effects of fried meat ingestion on DNA damage in lymphocytes and detect changes in urinary mutagenicity related to these dietary regimens.

Much of the experimental work examining the genotoxic effects of compounds in fried meat, particularly heterocyclic amines (HCAs), has been conducted in animal and *in vitro* systems. For example, numerous animal studies examining the genotoxic effects of exposures to single heterocyclic amines (HCAs) have been conducted, often with HCA doses exceeding 10 mg/kg/day. In contrast, human exposures to HCAs found in meat cooked at high temperatures have been estimated to be in the range of 200 ng/person/day to several µg/person/day (Turteltaub et al., 1997, Butler et al., 2003). Extrapolation of high-dose exposures in animals to the effects of low-dose, chronic exposures may be misleading. For example, results from a study comparing low-dose exposures to MeIQx in rodents and humans suggest that linear extrapolation from high-dose studies may underestimate damage in tissues from low-dose exposures (Turteltaub et al., 1997).

Previous controlled feeding studies in humans have focused on changes in urine mutagenicity after consumption of fried meat, or inhibitors of fried meat-induced mutagenesis (Baker et al., 1982, Dolara et al., 1984, Hayatsu et al., 1985, Ohyama et al., 1987, Hayatsu and Hayatsu, 1993, DeMarini et al., 1997, Gabbani et al., 1998, Murray et al., 2001, Peters et al., 2003). Sinha and colleagues (1994) have measured increased HCAs levels in urine and increased CYP1A2 enzyme activity after consumption of meat fried at high temperatures. However, measurements of urine mutagenicity reflect exposure but do not provide information on fried meat-induced damage in relevant tissues, e.g., colon epithelial cells.

Studies at Lawrence Livermore National Laboratory (LLNL) have examined the formation of DNA adducts in humans following the ingestion of radio-labeled HCAs, with doses of 228 µg [^14^C]-MeIQx and 70-84 µg [^14^C]-PhIP, respectively. These studies suggest that 1) both PhIP and MeIQx are bioavailable to the colon, and 2) that the human colon is more susceptible to the genotoxic effects of MeIQx than the rat colon (Turteltaub et al., 1997, Dingley et al., 1999). However, the doses used in these studies are one to two orders of magnitude higher than typical dietary exposures to HCAs.

Unlike the LLNL studies, in which subjects were fed purified HCAs, our study will focus on the effects of diets that are high in fried meat. As a result, subjects in our study will be exposed to concentrations of HCAs that are typical in Western diets compared with the relatively high concentrations used in the LLNL studies. Furthermore, our findings will be more readily interpretable with respect to the way people in free-living populations actually eat. Our study design is based on previous controlled feeding studies (Sinha et al., 1994, Peters et al., 2003). Our study will extend those findings by monitoring not only exposure to fried meat (urine mutagenicity) but also damage (comet measurements in lymphocytes and colon cells). Thus, results from our study may help to better characterize the risks of exposure to fried meat mutagens and the protective effects of inhibitors of those mutagens.

To our knowledge, there are no studies that have measured the kinetics of heterocyclic amines in blood following fried meat consumption. Mutagenicity of urine extracts from previous feeding studies may provide some information on the distribution and excretion of HCAs and HCA metabolites. In a study by Peters and colleagues (2003), urine mutagenesis peaked 2-6 hours after fried meat consumption followed by a decline in mutagenesis after 12 h. However, overall mutagenicity increased significantly over the 7-day feeding period, suggesting either an accumulation of mutagens or increased production of mutagenic metabolites. Sinha and colleagues (1994) observed increased CYP1A2 activity after consumption of meat fried at high temperatures over a one-week period. Thus, fried meat consumption induces enzyme activity that, in turn, produces increased levels of reactive metabolites. Exposure to mutagenic metabolites may increase with exposure time.

Based on the results of several animal and human experiments and *in vitro* studies, it appears that certain dietary components may help to ameliorate the effects of fried meat consumption *in vivo*. We will test the hypothesis that consumption of certain other foods and dietary constituents will ameliorate the short-term damaging effects associated with fried meat consumption. Specifically, we will determine whether consumption of fermented dairy products (i.e., yogurt), chlorophyllin (a salt of chlorophyll found in green plants), and cruciferous vegetables (e.g., cabbage, broccoli, and Brussels sprouts), will significantly lessen or “block” the deleterious action of heterocyclic amines on colonic epithelial cells and white blood cells and whether this diet will decrease urinary mutagenicity. We do not have specific information on the exposure time for the putative inhibitors to show a protective effect against heterocyclic amine-induced damage. However, in one human study, expression of phase II enzymes increased significantly after one week of consuming cruciferous vegetables (Nijhoff et al., 1995). Therefore, detoxification of fried meat mutagens may be enhanced after one week of cruciferous vegetable consumption.

Before undertaking the main study to test these hypotheses, we propose undertaking a pilot study to determine the magnitude of the effects that can be detected in colon cells and white blood cells after ingestion of fried meat and inhibitors of fried meat-induced DNA damage and the length of time these diets must be consumed in order for these effects to be detected. One of the aims of the pilot study is to determine whether a subject must be on a fried-meat diet or a fried-meat-plus-inhibitor diet one or two weeks before changes in colon epithelial cells can be observed. Cells within colon crypts turn over every 10-12 days (Twombly, 2002). Thus, two weeks may be the maximum exposure time before colon cells are replaced. In this study design, we will monitor DNA damage each week in the two-week feeding periods to help determine the optimal exposure time.

The results of this pilot study will provide information that will be used to determine the feasibility of design of a larger study. For example, such information will help to determine the sample size required in the larger study. The pilot study will also serve to assess the diet and activity questionnaire used in the study, as well as subject compliance with dietary regimens and physician appointments. Thus, the methods discussed below relate to the pilot study.

# Overview of Methods

The main goal of the study is to measure urine mutagenicity and short-term DNA damage in lymphocytes and colon cells associated with the consumption of fried meat and to determine whether any fried meat-induced mutagenicity and DNA damage that is detected can be inhibited by consumption of yogurt, cruciferous vegetables, and chlorophyllin tablets. DNA damage associated with the different dietary regimens will be assessed in the comet assay. This study will take place in four major parts, each of which will be discussed in detail below: 1) Participant enrollment; 2) Dietary regimens; 3) Sample collection and processing; 4) Statistical design and analysis.

# 1. Enrollment of Subjects

Subjects will be recruited through fliers, posted at UNC Hospitals and in buildings on the University Campus. A pre-screening interview will be conducted by telephone, and subjects meeting preliminary inclusion criteria for the study will be interviewed for a second time at the General Clinical Research Center (GCRC) at UNC Hospitals. Information collected in the initial telephone screening interview from individuals who do not meet the eligibility requirements or choose not to participate will be immediately destroyed. A total of 16 subjects, eight men and eight women, who meet the inclusion criteria, will be enrolled in the study. Due to space constraints at the GCRC, subjects will take part in the study in groups of four. We chose a sample size of 16 for the pilot study based on both the two-part study design, described below, and the limitation of enrolling four subjects at a time in the GCRC. In our proposed study design, subjects will be enrolled in “batches” of four, with two subjects assigned to each sequence. We will seek to address inter-individual differences, as well as period effects, by employing this study design.

#

#

# *Inclusion/Exclusion Criteria*

Nonsmoking, English-speaking, healthy adults (eight men and eight women), ages 18-45, will be enrolled. Pregnant women will be excluded, as the dietary regimen in this study is not optimal for pregnant women. Pregnancy tests will be administered to women at the beginning of the study and women will be encouraged to avoid pregnancy during the study. Individuals will also be excluded if they consume more than two alcoholic drinks per day, have a history of illicit drug use, have a history of goiter, diabetes, colitis, a diagnosed current thyroid condition, or occult bleeding, are excessively obese (BMI > 30), or are vegetarians. In addition, individuals on prescription medications or antibiotics will be excluded; antibiotics will interfere with laboratory assays used in this study. Individuals on anti-coagulant medication will also be excluded. Consumption of tea, or use of aspirin, nonsteroidal anti-inflammatory drugs (NSAIDs), or vitamin or herbal supplements by participants will also be prohibited during the study. We are excluding tea consumption during this study because of the possible confounding effects of compounds in tea. Catechins and polyphenols in tea may have an inhibitory effect on the damage induced by fried meat consumption. Alcohol consumption will be prohibited during the study. We will allow subjects to consume limited amounts of coffee and diet cola during this study. Because this study is not specifically related to childhood disease, children will not be enrolled.

**2. Dietary regimens**

At the beginning of the study, subjects will be instructed to consume only the foods provided by the GCRC throughout the study period. Before beginning the feeding portion of the study, participants will be asked to complete a food frequency questionnaire to provide estimates of previous dietary exposure that may affect outcomes in this controlled feeding study. The Study Coordinator will conduct the pre-screening interview (to determine eligibility for the study) and the diet and activity questionnaire. The Research Nutritionist will perform the Screening Interview and diet instruction for subjects enrolled in the study.

Daily visits to the GCRC for breakfast and dinner will be required except on Saturday and Sunday (see Table 1). On Friday of each week during the study period, subjects will be provided boxed lunches and breakfasts, but will be required to consume evening meals on Saturdays and Sundays at the GCRC. GCRC staff will monitor evening meals to insure that participants are consuming the evening meals. All meals will be prepared at the GCRC, taking into account subjects’ food preferences.  The diets will be isocaloric, with the daily calorie intake based on each subject's predicted metabolic demand relative to body weight.  Subjects will be weighed daily and calorie levels will be adjusted when a weight gain or loss is observed over a 7-day period.

|  | Fri. | Sat. | Sun. | Mon. | Tues. | Wed. | Thurs. | Fri. | Sat. |
| --- | --- | --- | --- | --- | --- | --- | --- | --- | --- |
| Meals | Breakfast  GCRC | Packed  Breakfast | Packed  Breakfast | Breakfast  GCRC | Breakfast  GCRC | Breakfast  GCRC | Breakfast  GCRC | Breakfast  GCRC | Breakfast  GCRC |
|  | Boxed Lunch | Boxed Lunch | Boxed  Lunch | Boxed Lunch | Boxed  Lunch | Boxed Lunch | Boxed  Lunch | Boxed Lunch | Boxed Lunch |
|  | Dinner  GCRC | Dinner  GCRC | Dinner  GCRC | Dinner  GCRC | Dinner  GCRC | Dinner  GCRC | Dinner  GCRC | Dinner  GCRC | Dinner  GCRC |
| Urine Collection | Overnight  Urine (First- Morning Void) |  |  | Overnight  Urine | Overnight  Urine | Overnight  Urine | Overnight  Urine | Overnight  Urine | Overnight  Urine |
| Physician Appointment |  |  |  |  |  |  | Blood Draw  and  Rectal  Biopsies |  |  |

# Table 1. Feeding schedule for study participants.

Of the 16 participants enrolled in the pilot study, eight (four men and four women) will be randomly assigned to Part A of the study design, with two men and two women randomly assigned to each sequence (Fig. 1). In sequence 1, subjects will be fed a baseline diet (15% protein, 55% carbohydrates, and 30% fat) for two weeks. Any meat in the baseline diet will be prepared in a way that produces low or non-detectable levels of heterocyclic amines. Fermented milk products and cruciferous vegetables will be excluded from the baseline diet. After consuming the baseline diet for two weeks, subjects will be fed the baseline diet but with the addition of lean ground beef (15% fat), pan-fried at 250° C for 11 min per side, or a total of 22 min (this diet will also contain 15% protein, 55% carbohydrates, and 30% fat). The amount of meat consumed by each subject will depend on body weight, equivalent to 3.1-4.4 g/kg of body weight, or 200g meat/day for a 64.5 kg person. In sequence 2, subjects will be fed the baseline diet along with fried meat for two weeks followed by a two-week baseline only diet. The purpose of this crossover design is to eliminate confounding of treatment effects with period effects that would occur if all eight subjects were assigned to a single sequence.

**Sequence 2**

Baseline Diet

Baseline Diet

Baseline Diet

Baseline Diet

Baseline Diet

plus Fried Meat

Baseline Diet

plus Fried Meat

Baseline Diet

plus Fried Meat

Baseline Diet

plus Fried Meat

**Sequence 1**

Week 1

Week 2

Week 3

Week 4

Figure 1. Study Design for the Pilot Study (Part A) to examine the effect of fried meat on DNA in colon cells and lymphocytes, and effects on mutagenicity in urine.

Following the completion of Part A of the study design, which compares the effects of fried meat to baseline diet, Part B of the study design will be undertaken to determine whether consumption of yogurt, cruciferous vegetables, and chlorophyllin tablets along with fried meat will ameliorate any transient damaging effects of fried meat. The remaining eight subjects will be randomly assigned to Part B, with two men and two women randomly assigned to each sequence (Fig. 2). In sequence 1, subjects will be fed the baseline diet along with fried meat over a two-week period. In the second two-week period, the subjects will be fed the baseline/fried meat diet along with additional components consisting of: 500 g/day of cruciferous vegetables, including broccoli, cauliflower, Brussels sprouts, and cabbage, yogurt fermented with *S. thermophilus*, *L. bulgaricus*, and *L. casei* containing at least 10^10^ CFU/ml of lactic acid bacteria strains each day, and one tablet containing 100 mg sodium copper chlorophyllin (Rystan Inc., Little Falls, NJ) three times a day. We have applied to use the Investigational Drug Service at UNC Hospitals in this study in order to dispense chlorophyllin (Derefil) tablets.

This diet will also contain 15% protein, 55% carbohydrates, and 30% fat. In sequence 2, subjects will be fed the diet that includes the additional inhibitor components during the first two weeks and the baseline/fried meat diet during the second two weeks.

**Sequence 2**

Baseline Diet

plus Fried Meat

Baseline Diet

plus Fried Meat

Baseline Diet

plus Fried Meat

Baseline/Fried Meat

plus Inhibitors

Baseline/Fried Meat

plus Inhibitors

Baseline/Fried Meat

plus Inhibitors

**Sequence 1**

Baseline Diet

plus Fried Meat

Baseline/Fried Meat

plus Inhibitors

Figure 2. Study Design for the Pilot Study (Part B) to examine the putative inhibitors of transient DNA damage associated with fried meat consumption in colon cells and lymphocytes, and effects on mutagenicity in urine.

**3. Sample collection and processing**

Urine samples, stool samples, blood samples and rectal biopsies will be taken from subjects at the GCRC as described below. Details of sample collection and processing are shown in Figure 3. Unused samples of blood, urine, and tissue will stored for future use, which may include genetic research. In addition, a sample of fried meat used in the study will be tested for heterocyclic amine content as described in Appendix A.

# *Urine Samples*

In this study, subjects will be asked to collect urine samples (first-morning void), which will be used to determine changes in mutagenicity associated with different dietary regimens. Each evening, Sunday through Thursday, subjects will be provided with sterile specimen jars labeled with the subject’s study code and will be instructed to collect approximately 60 cc of first-morning void urine. When subjects report to the GCRC for breakfast they will give the urine sample to a member of the GCRC nursing staff who will store the samples at -20ºC at the GCRC. On Thursday of each week of the study, CODA personnel will collect all urine samples from the preceding study week, transport the samples to the CODA facility, and document receipt of all samples. Samples will be stored at -80ºC at the CODA facility. Portions of the urine sample collected on Thursday mornings of each week of the study will then be sent to the U.S. Environmental Protection Agency for mutagenicity testing (see Appendix A for details). The remaining urine samples collected on previous study days will be stored for future unspecified medical research.

*Stool Samples*

Subjects will be provided with plastic containers lined with a plastic bag and asked to collect a stool sample during the second and fourth weeks of the study. The first stool sample will be collected on day 12-14, and the second will be collected on day 26-28. Samples will be collected by a member of the GCRC nursing staff who will store the samples at -20ºC at the GCRC. On Thursday of weeks 2 and 4 of the study, CODA personnel will collect all stool samples, and transport the samples to the CODA facility. Samples will be lyophilized and stored at -80ºC at the CODA facility.

*Blood Samples*

On Thursdays of each week of the feeding study, appointments will be made with the gastroenterologist (Dr. Lisa Gangarosa) for the purpose of obtaining blood samples and rectal biopsies from study subjects. These appointments will last approximately 30 minutes (10 minutes for the blood draw, 20 minutes for the biopsy). Approximately 24 cc of blood from each subject will be collected; 18 cc will be collected in 3 yellow-top tubes (Becton, Dickinson and Company) and 6 cc will be collected in one red-top tube. The blood will be stored at room temperature. One yellow-top tube from each subject will be received by CODA personnel and transported directly to NIEHS laboratories for processing and use in the comet assay (see Appendix B for details). Blood collected in the remaining two ACD tubes and red-top tube will be will be transported to the CODA facility by CODA personnel, catalogued, and processed for storage.

*Rectal Biopsies*

During the Thursday appointment with Dr. Gangarosa, four pinch biopsies will be taken from the rectum of each subject. Specimens will be obtained in patients who have not had any enemas or other preparation. Patients will not be sedated. Dr. Gangarosa, who is trained in this procedure, will collect the biopsies in the General Clinical Research Center. The biopsies will be obtained using a disposable, fenestrated, spiked, flexible biopsy forceps (Boston Scientific) mounted on a semi-rigid rod through a short (25 cm) rigid disposable (plastic) sigmoidoscope. The patient will be placed in the left lateral decubitus or Sims’ position. The sigmoidoscope will be lubricated and inserted blindly with the obturator in place for 3-5cm. The obturator will be removed and instrument inserted to 10 cm. The biopsy specimens (“non-prep” biopsies) will be taken under direct vision from the mucosa of a valve of Houston in the rectum 10 cm above the level of the anal verge. Care will be taken to place the fully open biopsy forceps at right angles to and against the mucosa in order to get a sample of sufficient area and depth. At least four adequate biopsies will be obtained. If a biopsy pinch is inadequate, another will be obtained. The biopsy specimen will be removed from the forceps by the Project Manager who will gently tease it onto a strip of bibulous paper provided by the laboratory. Biopsies will be transferred to sterile 13 ml tubes containing Hank’s Buffered Saline Solution containing 0.3% bovine serum albumin and 1% penicillin/streptomycin (HBSSsup) and transported on ice to NIEHS for processing. Samples will be processed within two hours of the biopsy procedure (for details see Appendix B).

***Disposition of Stored Samples/Data***

Access to stored samples will be limited using a locked room. Samples and data will be stored using codes assigned by the investigators. Data will be kept in password-protected computers. Only investigators will have access to the samples and data.

In the future, other investigators (both at NIH and outside) may wish to study these samples and/or data. In that case, IRB approval must be sought prior to any sharing of samples. Any clinical information shared about the sample would similarly require prior IRB approval.

Any loss or unanticipated destruction of samples or data (for example, due to freezer malfunction) that meets the NIH Intramural Protocol Violation definition or results in a violation that compromises the scientific integrity of the data collected for the study, will be reported to the NIEHS IRB.

At the completion of the protocol (termination), samples and data will either be destroyed or, after IRB approval, transferred to another existing protocol, in which case the samples will be maintained in a repository.

Additionally, subjects may decide at any point not to have their samples stored. In this case, the principal investigator will destroy all known remaining samples and report what was done to both the subject and the IRB. This decision will not affect the subject’s participation in this protocol or any other protocols at NIH.

**4. Statistical design and analysis**

We chose a cross-over design for this pilot study to eliminate confounding between period and treatment effects and to enable us to make treatment effects comparisons within subjects rather than between subjects. The former consideration should reduce possible bias and the latter consideration should enhance the precision of our estimates of treatment effects. For parts A and B of the pilot study, half the subjects on each sequence will be men and half women. The slots available to each sex in each sequence within each part will be randomly assigned to the 8 men and 8 women recruited. The particular cross-over design chosen involves four one-week periods, with each dietary regime assigned to two consecutive weeks. By allowing two consecutive weeks on each diet, the design for the pilot study will help us to make an informed judgment about the whether a subject must be on a fried-meat diet or a fried-meat-plus-inhibitor diet one or two weeks before changes in colon epithelial cells can be observed. This information will be critical in the design of the main study.

The four primary outcomes to be analyzed statistically are the results of the urinary mutagenicity assay, the levels of heterocyclic amines in urine, and the comet assay results for lymphocytes and for colon epithelial cells. The first of these, urine mutagenicity, is, at its base, a count of mutant bacterial colonies and may be analyzed as essentially a Poisson random variable, perhaps allowing extra-Poisson variation. The last three outcomes can be regarded as continuous random variables. Concentrations of heterocyclic amines will be log-transformed and analyzed as approximately normally distributed. For the comet assays, four aliquots of cells from each sample are placed on microscope separate slides and assayed. Olive tail moment (OTM), a measure of DNA damage, is assessed for 25 cells selected at random on each slide and median for each slide is determined. The average of the logarithms of the four medians provides a summary measure of DNA damage for each sample that can be analyzed as approximately normally distributed.

Cross-over designs with continuous outcomes have traditionally been analyzed using ordinary least squares techniques or using paired tests of selected within-subjects contrasts to study hypotheses of interest about direct and carry-over treatment effects. More recently, mixed model approaches to such analyses have become popular. We intend to use a mixed model analysis (generalized linear mixed model for the approximately Poisson outcome) but recognize that, with only eight subjects per sub-study, benefits from formally modeling subjects as random effects may be minimal. Because we view the proposed study as a pilot study, designed to gather additional quantitative information to aid in the design of a subsequent clinical study rather than to yield definitive results about hypotheses of interest, we have not investigated the power of the proposed study for detecting direct or carry-over treatment effects

**Urine**

(First morning void collected by the patient before coming to the GCRC)

**Blood**

(Drawn at GCRC into 3 yellow-top tubes and 1 red-top tube)

**Rectal Biopsies**

(Biopsies obtained at the GCRC)

Urine specimens collected from the previous week (stored at -20° C) picked up by CODA personnel and transported to CODA and stored at -80° C

Portion (40-50 ml) of the Thurs. a.m. urine sent to the USEPA in RTP for mutagenicity assay

Red-top tube and 2 yellow-top tubes transported by CODA to CODA facility for processing and storage

1 yellow-top tube transported to NIEHS by CODA for processing within 2 hours of blood draw

Lymphocytes isolated from whole blood used in the comet assay

Biopsies transferred to 13 ml tubes containing HBSS supplemented with FBS and penicillin/streptomycin

Biopsies transported to the NIEHS by CODA personnel for processing

Colon cells isolated from biopsies used in the comet assay

**Stool**

(Collected in the 2^nd^ and 4^th^ weeks of the study)

Stool samples (stored at -20° C) picked up by CODA personnel and transported to CODA and stored at -80° .

Figure 3. Collection, transport and processing of samples obtained from subjects on Thursdays of the feeding study

Appendix A

# Mutagenicity of Cooked Meat and Urine Samples

# Mutagenic activity of the fried hamburger used in this study will be determined in the *Salmonella* strain YG1024 (*hisD3052*, *rfa*, *uvrB*, pKM101, pYG219) in the presence of araclor-induced S9 rat liver homogenate (2 mg of S9 protein/plate) in the standard plate incorporation assay. Heterocyclic amine content in meat samples will be analyzed using the solid phase extraction and HPLC method. Mutagenicity of urine samples will also be determined in the *Salmonella* strain YG1024. Approximately 60 ml of the urine samples collected on Thursdays (after a complete week of each feeding regimen) will be extracted using C18 resin columns and methanol. A portion of this extract will be solvent-exchanged into dimethyl sulfoxide (DMSO) at a 150X concentration for use in the mutagenicity assay. This portion represents the unhydrolyzed or free urinary mutagencity. The remaining portion of the extract will be dissolved in water and hydrolyzed at 70°C in 6 N HCL. This portion represents the hydrolyzed urinary mutagenicity, which is the combination of free and conjugated mutagenicity, e.g., heterocyclic amines that have been conjugated by metabolizing enzymes. Extracts of urine will be tested in 0.5, 1, 3 7, and 14 ml-equivalents /plate in the *Salmonella* strain YG1024 in the presence of araclor-induced rat liver S9 mix (2 mg of S9 protein/plate). Depending on the amounts of urine extracts recovered from each subject, urine mutagenicity will be determined in duplicate or triplicate plates for each sample. Mutagenic potencies will be expressed as revertant colonies per ml-equivalents of urine.

# Appendix B

# Processing blood for use in the comet assay

# Blood collected in yellow-top tubes will be transferred to ACCUPIN tubes (Sigma) and centrifuged at 800 x *g* for 15 minutes at 26° C. Mononuclear cells (primarily lymphocytes), which form an opaque layer after centrifugation, are removed, washed with Phosphate Buffered Saline (PBS) solution, and resuspended in PBS at a density of 10^6^ cells/ml. Approximately 20,000 cells in 10 l aliquots are pipetted along with low melting point agarose onto glass slides that have been coated with 1% normal melting point agarose. Slides are refrigerated at 4° C to allow the agarose to solidify. A second layer of low melting point agarose (100l) is added to the slides and solidified at 4° C. Slides are placed in lysis buffer (NaCL, Na_2_EDTA, Trizma Base, DMSO, and Triton-X 100) overnight. Slides are then neutralized (.4 M Tris, pH 7.5) for 5 minutes and placed in an electrophoresis chamber containing electrophoresis buffer (10N NaOH and 200mM EDTA pH>13.0) for 20 minutes to allow for unwinding of DNA and then are subjected to electrophoresis for 40 minutes (25 V, 300 mA). After electrophoresis, slides are washed with neutralization buffer and placed in ice-cold ethanol for at least 1 hour. To visualize damage in individual cells, slides are stained with ethidium bromide and visualized using a fluorescent microscope. Damage will be quantitated using Komet 5.0 (Kinetic Imaging) software. For each sample, four slides will be prepared and 25 comets will be scored for each slide, for a total of 100 comets for each sample.

# Isolation of colon cells from rectal biopsies for use in the comet assay

To isolate colon epithelial cells, biopsy specimens will be minced with fine scissors and incubated in 3 ml of HBSS supplemented with 6 mg of proteinase K and 3 mg of collagenase for 1 hour in a shaking water bath at 37 ° C. The cell suspension is then diluted to a volume of 15 ml and centrifuged at 139 x *g* for 6 minutes. Pellets are resuspended in PBS at a density of approximately 10^6^ cells/ml. Approximately 20,000 cells in 10 l aliquots are pipetted onto glass slides for use in the Comet Assay as described previously. Four slides will be prepared for each specimen, 25 individual cells from will be scored for damage for a total of 100 cells for each sample.

# Selected References

Baker, R., Arlauskas, A., Bonin, A. and Angus, D. (1982) *Cancer Lett,* 16, 81-9.

Butler, L. M., Sinha, R., Millikan, R. C., Martin, C. F., Newman, B., Gammon, M. D., Ammerman, A. S. and Sandler, R. S. (2003) *Am J Epidemiol,* 157, 434-45.

DeMarini, D. M., Hastings, S. B., Brooks, L. R., Eischen, B. T., Bell, D. A., Watson, M. A., Felton, J. S., Sandler, R. and Kohlmeier, L. (1997) *Mutat Res,* 381, 83-96.

Dingley, K. H., Curtis, K. D., Nowell, S., Felton, J. S., Lang, N. P. and Turteltaub, K. W. (1999) *Cancer Epidemiol Biomarkers Prev,* 8, 507-12.

Dolara, P., Caderni, G., Salvadori, M., Tringale, L. and Lodovici, M. (1984) *Cancer Lett,* 22, 275-80.

Gabbani, G., Nardini, B., Bordin, A., Pavanello, S., Janni, L., Celotti, L. and Clonfero, E. (1998) *Mutagenesis,* 13, 187-91.

Hayatsu, H. and Hayatsu, T. (1993) *Cancer Lett,* 73, 173-9.

Hayatsu, H., Hayatsu, T. and Ohara, Y. (1985) *Jpn J Cancer Res,* 76, 445-8.

Murray, S., Lake, B. G., Gray, S., Edwards, A. J., Springall, C., Bowey, E. A., Williamson, G., Boobis, A. R. and Gooderham, N. J. (2001) *Carcinogenesis,* 22, 1413-20.

Nijhoff, W. A., Mulder, T. P., Verhagen, H., van Poppel, G. and Peters, W. H. (1995) *Carcinogenesis,* 16, 955-7.

Ohyama, S., Kitamori, S., Kawano, H., Yamada, T., Inamasu, T., Ishizawa, M. and Ishinishi, N. (1987) *Mutat Res,* 192, 7-10.

Peters, U., Sinha, R., Bell, D. A., Rothman, N., Grant, D. J., Watson, M. A., Kulldorff, M., Brooks, L. R., Warren, S. H. and DeMarini, D. M. (2003) *Environmental and Molecular Mutagenesis,* 43, 53-74.

Sinha, R., Rothman, N., Brown, E. D., Mark, S. D., Hoover, R. N., Caporaso, N. E., Levander, O. A., Knize, M. G., Lang, N. P. and Kadlubar, F. F. (1994) *Cancer Res,* 54, 6154-9.

Turteltaub, K. W., Mauthe, R. J., Dingley, K. H., Vogel, J. S., Frantz, C. E., Garner, R. C. and Shen, N. (1997) *Mutat Res,* 376, 243-52.

Twombly, R. (2002) *Environ Health Perspect,* 110, A394-5.
